# Uncovering the glutamate carboxypeptidase II microenvironment using a multi-labeling proteomic approach

**DOI:** 10.1038/s41598-025-28826-5

**Published:** 2025-11-27

**Authors:** Jana Pokorná, Martin Hadzima, Alena Křenková, Joshua D. Smith, Vladimír Šubr, Robin Kryštůfek, Martin Hubálek, Jana Starková, Karolína Šrámková, Tomáš Etrych, Libor Kostka, František Sedlák, Jan Konvalinka, Pavel Šácha

**Affiliations:** 1 Institute of Organic Chemistry and Biochemistry, Czech Academy of Sciences, Flemingovo nám 542/2, Prague, 160 00 Czech Republic; 2 Department of Biomedical Polymers Institute of Macromolecular Chemistry, Czech Academy of Sciences, Heyrovského nám 2, Prague, 160 00 Czech Republic; 3 Department of Biochemistry and Experimental Oncology, First Faculty of Medicine, Charles University, U Nemocnice 5, Prague, 128 00 Czech Republic

**Keywords:** PSMA, Protein proximity labeling, iBody, HRP, µMap, Riboflavin, Biochemistry, Biological techniques, Biomarkers, Cancer, Computational biology and bioinformatics

## Abstract

Glutamate carboxypeptidase II (GCPII) is a membrane-bound metallopeptidase predominantly expressed in neural and prostatic tissues, with significantly elevated levels in prostate carcinoma that increase with tumor grade. Despite its significance as a target for imaging and therapy in prostate cancer, its physiological function in this tissue remains poorly understood. To help fill this knowledge gap, we developed an integrated approach combining proximity labeling technologies for proteomic profiling (horseradish peroxidase, µMap, and riboflavin tetraacetate labeling) with our previously established iBody platform, which targets GCPII with a small-molecule specific inhibitor with proven efficacy as a chemical probe. Proximity labeling proteomic experiments on U251 MG–GCPII cells were followed by mass spectrometry and statistical analysis of protein abundances obtained by label-free quantification. Additionally, selected identified proteins were further validated through Western blot analyses and GCPII pulldown assays using cell lysates. This work identifies a network of GCPII-associated proteins that are potentially involved in cancer metabolism, migration, invasiveness, progression, and immune evasion. Our novel proximity proteomics labeling strategy provides a low-background framework, efficient biotinylation, and enhanced target binding via the avidity effect. Among the approaches tested, riboflavin tetraacetate-based iBody labeling exhibited the highest precision, underscoring its potential for membrane protein interactome mapping.

**Supplementary Information:**

The online version contains supplementary material available at 10.1038/s41598-025-28826-5.

## Introduction

Glutamate carboxypeptidase II (GCPII), also known as prostate-specific membrane antigen (PSMA) in the context of solid tumor microenvironments, is a membrane zinc metalloprotease found in a broad range of tissues, with function dependent on its localization. The expression and function of GCPII are best characterized in the central nervous system (CNS) and the jejunum, where the enzyme regulates the level of neuropeptide N-acetyl-aspartyl-glutamate (NAAG)^1^and hydrolyzes dietary folates^2^, respectively.

In addition to the healthy tissues, GCPII is present in prostate adenocarcinoma, with expression levels correlating with cancer grade^3,4^. It is also expressed in various prostate cancer metastases^5^ and in the neovasculature of most human solid tumors^6^. These characteristics make GCPII an outstanding target not only for imaging prostate cancer lesions by PET or SPECT^7^ but also for systemic therapy. Pluvicto^®^(lutetium Lu 177 vipivotide tetraxetan), a radioligand therapy, was approved by the FDA in 2022 for the treatment of patients with advanced prostate cancer^8,9^.

However, in the prostate, kidneys, and solid-tumor environments, the precise role of GCPII remains poorly understood. Because localization of proteins is often connected with their function^10^, understanding the GCPII microenvironment may provide insights into its function(s) in these tissues. To uncover the cryptic role of GCPII, we leveraged proximity proteomics to investigate its surrounding protein environment.

Proximity proteomics has become an increasingly popular tool to probe small–molecule-protein and protein-protein interactions. Proximity investigations leverage different labeling methods by tagging interacting or proximal proteins. Traditionally, labeling methods have been enzyme-derived, including BioID^11^, ascorbate peroxidase (APEX)^12^, and horseradish peroxidase (HRP)^13,14^. Modern labeling methods have expanded to exploit low-molecular labeling catalysts, such as iridium complexes (µMap)^15^ or riboflavin^16,17^. All the above mentioned methods rely on covalent attachment of biotin to the labelled protein, enabling the enrichment of tagged proteins via streptavidin affinity purification^18^. Enriched samples are digested and analyzed bottom-up by high-resolution mass spectrometry coupled to a nano-flow liquid chromatography system, followed by subsequent data analysis using proteomic software^19^. The methods differ in the nature of the catalyst as well as the reactive species utilized for covalent labeling which influences the labeling radius of the biotinylating platform. Iridium-catalyst labeling exploits conversion of diazirine to a highly reactive carbene species^15^, while HRP and riboflavin utilize phenoxyl radicals for covalent tagging^14,17^. The HRP labeling radius using biotinyl-tyramide, as measured by a super-resolution microscopy-based assay, is 269 ± 41 nm^20^, BioID with biotin has been reported to achieve approximately 10 nm^21^, and iridium-catalyst labeling was reported to achieve a radius of less than 4 nm^15^. The exact radius of riboflavin-derived labeling has not been determined, but is expected to be smaller than those of its enzyme counterparts due to its compact low-molecular nature^17^.

Each proximity labeling method yields distinct labeling profiles and can be strategically selected depending on the biological question. However, these methods require careful optimization for specific applications and are sometimes limited depending on the interactome. For example, HRP and BioID methods are restricted to protein-protein interactions due to the size of catalysts themselves and the need to attach a small molecule for direct protein targeting^11,14^. Polymer offers a unique platform to alleviate problems associated with labeling-machinery size by providing a highly tunable backbone allowing for the combination of protein-tagging machinery and targeting small molecules^22^. iBodies, antibody mimetics based on the stable, biocompatible, non-toxic, and non-immunogenic copolymer based on *N*-(2-hydroxypropyl)methacrylamide (pHPMA), offer a modular system that enables controlled, multivalent conjugation with diverse functional components^23–26^. We leveraged our established iBodies that are highly specific for GCPII^23,27^ to investigate and map the GCPII microenvironment by creating a novel platform that combines proximity proteomics with targeted iBodies.

## Results and discussion

### Preparation of iBodies

**Fig. 1:**
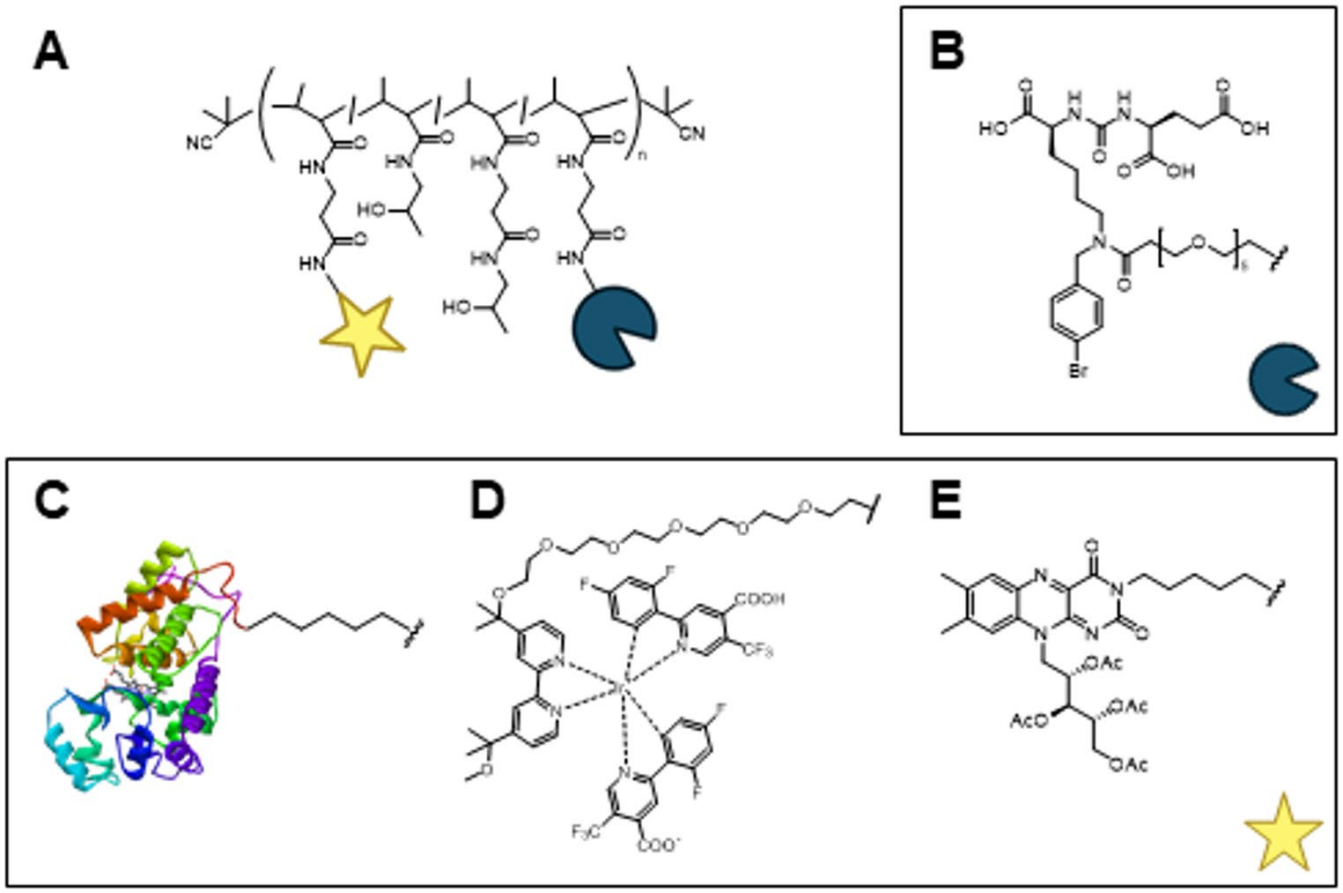
Schematic representation of iBodies. (**A**) HPMA copolymer decorated with a targeting ligand (blue circle) and a biotinylating moiety (yellow star). (**B**) GCPII inhibitor used as the targeting ligand for iBodies 1–5. (C-E) Biotinylating moieties: (**C**) HRP (N-terminally connected to iBody 1), (**D**) Ir cat (N-terminally connected to iBody 2), (**E**) RFT (iBody 3).

We generated three iBodies that incorporate a high-affinity small-molecule GCPII inhibitor (xGCPII, described in Šácha et al.^23^ as compound 1) and enable different labeling strategies using: **HRP** as a representative enzyme-based method, an iridium catalyst (**Ir cat**) or riboflavin tetraacetate (**RFT**) as recently developed light-mediated labeling approaches (Fig. 1A). These components were modified with either a PEG5 linker (for xGCPII, **HRP**, and **Ir cat**) or a C_6_ linker (for **RFT**) to reduce steric hindrance between the targeted and proximal proteins and the pHPMA backbone. After optimizing the experimental conditions, we evaluated the resulting iBodies for their effectiveness in tagging GCPII on the surface of live human glioblastoma cells stably transfected with GCPII (U251 MG–GCPII cell line). These cells were generated from the parental U251 MG line (provided by ATCC as U373 MG) using the Tet-Off Advanced System, which allows switchable GCPII expression, as previously described^28^. Copolymer precursors **P1** – **P3** for the construction of **iBodies 1–3** (pHPMA-co-Ma-*β*-Ala-TT) were synthesized via reversible addition–fragmentation chain transfer (RAFT) copolymerization of HPMA and Ma-*β*-Ala-TT monomers. Subsequently, after the conjugation of xGCPII (Fig. 1B), azide-modified **HRP** or **Ir cat** was linked to DBCO-functionalized iBody precursors **P1** or **P2**, respectively, yielding **HRP iBody 1** and **Ir cat iBody 2** (Fig. 1C, D). **RFT iBody 3** was synthesized through nucleophilic substitution, with the amine group of **RFT** reacting with TT moieties on the precursor of iBody 3 containing xGCPII (**P3**) (Fig. 1E). Similarly, nucleophilic substitution of TT groups was used to prepare additional constructs, including **iBody 4** (xGCPII and ATTO 488), designed for confocal microscopy, **iBody 5** (xGCPII and biotin), and **iBody 6** (biotin) for pulldown assay (Supplementary Table S1).

### PitStop 2 prevents GCPII internalization following iBodies binding

Due to the GCPII internalization upon ligand binding^29^, we tested three inhibitors of endocytosis: PitStop 2 (a clathrin-mediated endocytosis inhibitor), monodansyl cadaverine (MDC, a transglutaminase inhibitor), and LY 294002 (a broad-spectrum phosphoinositide 3-kinase inhibitor), in order to limit the labeling to the cell surface. We incubated the inhibitors with **iBody 4** (xGCPII and ATTO 488) and measured the iBody’s localization on U251 MG–GCPII cells using confocal microscopy (Supplementary Fig. S1). Following a 1–hour incubation, **iBody 4** bound to GCPII remained predominantly membrane-localized in the presence of PitStop 2, whereas MDC and LY 294002 failed to prevent internalization. Therefore, all subsequent experiments were conducted in the presence of PitStop 2.

### Optimization of biotinylation using RFT iBody 3

At the time of our study, HRP- and µMap-based labeling methods were already well-described in the literature, whereas biotinylation via RFT activation had only been briefly introduced by R. Oslund at the EFMC/ASMC19 symposium^16^. To optimize RFT-based labeling conditions, we conducted labeling experiments on U251 MG–GCPII cells with varying irradiation times using **RFT iBody 3** and biotinyl-tyramide. Following cell lysis and protein enrichment on streptavidin beads, we performed Western blot analysis of the resulting enriched proteins. We observed a progressive increase in GCPII biotinylation with longer exposures to 400 nm irradiation, plateauing at 5 min (Supplementary Fig. S2). This irradiation time was used in subsequent experiments. HRP- and µMap-based labeling were performed as previously described^14,15^.

### PPL of the GCPII microenvironment on live U251 MG–GCPII cells

Next, we performed iBody-targeted biotinylation using biotinyl-tyramide (for **HRP iBody 1** and **RFT iBody 3**) and biotinyl-PEG-5-diazirine (for **Ir cat iBody 2**) on live cells. U251 MG–GCPII cells were treated with 50 nM **HRP iBody 1**, **Ir cat iBody 2**, or **RFT iBody 3** in the presence of PitStop 2 for 20 min before adding the respective biotin substrate. Activation was induced by 0.03% H_2_O_2_ (**HRP iBody 1** labeling, 5 min), 450 nm irradiation (**Ir cat iBody 2** labeling, 10 min), or 400 nm irradiation (**RFT iBody 3** labeling, 5 min) (Supplementary Fig. S3). Control reactions included 2 mM 2-(phosphonomethyl)pentanedioic acid (2-PMPA), a specific GCPII inhibitor that competes with iBodies for binding to GCPII on the cell surface. After labeling, cells were lysed, and labeled proteins were enriched via streptavidin-bead pulldown. Protein eluants after streptavidin-bead enrichment were analyzed by Western blotting and mass spectrometry. Significantly altered proteins were determined by label-free quantitative proteomics, with the false discovery rate (FDR) set to 1%.

### RFT-based iBody labeling achieves superior Spatial precision

**Fig. 2:**
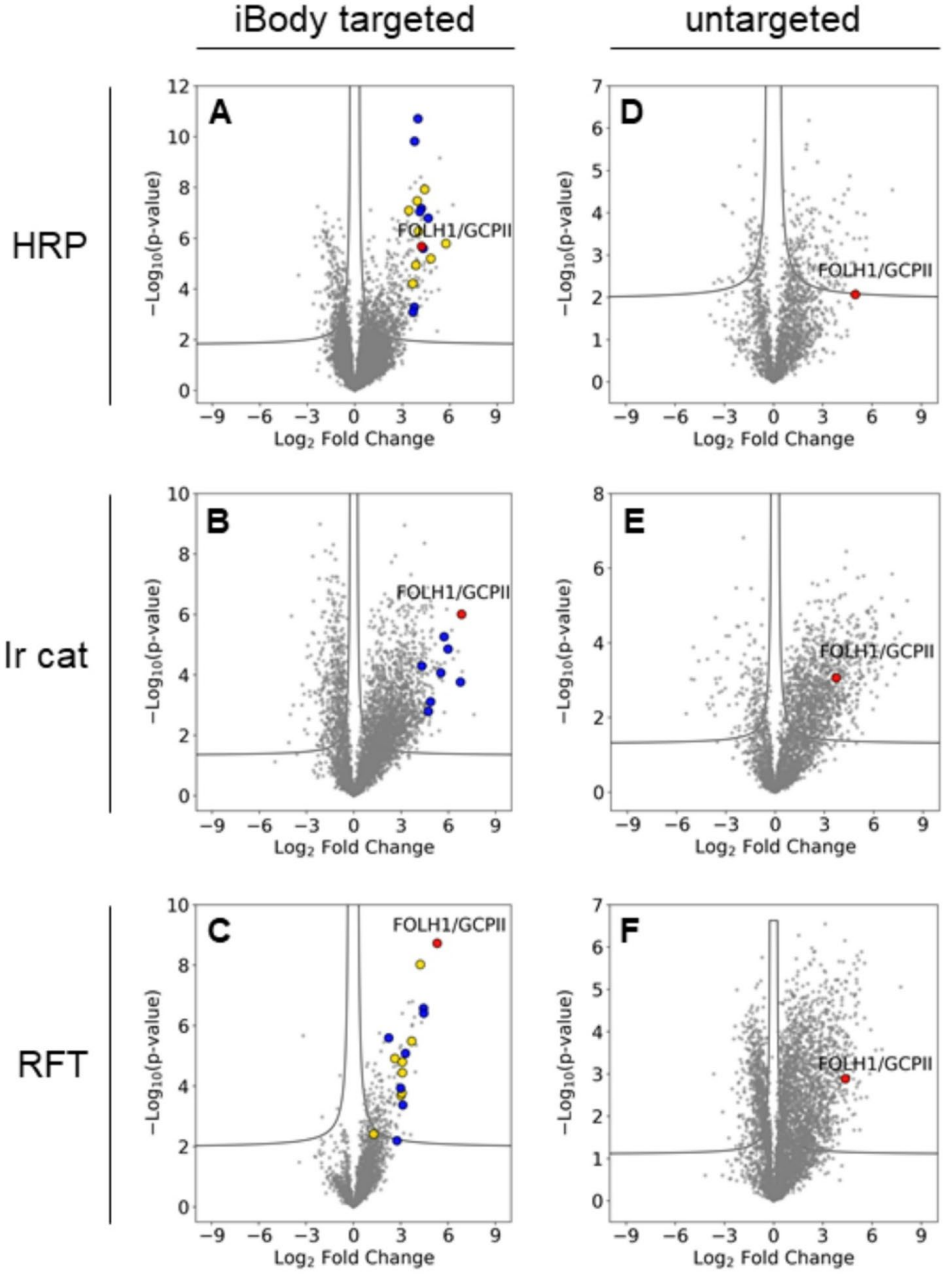
PPL of the GCPII microenvironment on live U251 MG–GCPII cells. (**A–C**) Quantitative proteomics volcano plots highlighting significantly enriched proteins (FDR–corrected *P* < 0.01). Averaged log₂ enrichment ratios [GCPII-targeted biotinylation by **HRP iBody 1** (A), **Ir cat iBody 2** (**B**), or **RFT iBody 3** (**C**) vs. reactions in the presence of the competitive inhibitor 2-PMPA] are plotted on the x-axis (*n* = 6 replicates), and –log₁₀ *P* values are on the y-axis. GCPII is highlighted in red, proteins validated via Western blot are shown in yellow, and those later confirmed by GCPII pulldown from cell lysate (without prior biotinylation) are marked in blue. (**D–F**) Untargeted labeling using free unconjugated catalysts. Volcano plots show averaged log₂ ratios [(**D**) free **HRP**, (**E**) free **Ir cat**, and (F) free **RFT** biotinylation vs. reactions in the absence of H_2_O_2_ (**D**) or irradiation (**E**,** F**)] on the x-axis (*n* = 3 replicates) and –log₁₀ P values on the y-axis. GCPII is highlighted in red. Untargeted catalysts failed to resolve GCPII from other proteins.

Proteomic analyses revealed that **HRP iBody 1** labeling resulted in a more extensive total protein enrichment profile than **RFT iBody 3** labeling, which is consistent with the broader labeling radius reported for the enzyme-based method^15,17^. **HRP iBody 1** labeled GCPII with relatively small significance (Fig. 2A) compared to **Ir cat iBody 2** where GCPII was identified as one of the most significantly enriched proteins, showing both a high P-value and fold change compared to the control on the volcano plot (Fig. 2B). Even higher GCPII- labeling specificity was achieved with **RFT iBody 3**; GCPII was the most enriched protein, suggesting highly precise, short-range labeling (Fig. 2C). This is further supported by the substantial percentage overlap between the proteins identified by the RFT method and those detected by the other two approaches. Specifically, the significantly enriched proteins identified by RFT showed an 85% overlap with proteins detected by at least one of the other two methods, whereas the HRP-identified proteins exhibited a 43% overlap and the Ir cat-identified proteins only a 13% overlap. We therefore believe that, although the RFT method identified significantly fewer proteins than the other two approaches, these proteins appear to be more relevant than the larger sets obtained using the HRP and Ir cat methods. Untargeted delivery of free unconjugated **HRP**, **Ir cat**, or **RFT** to live cells, followed by activation, failed to distinguish GCPII from other proteins (Fig. 2D-F). Surprisingly, **Ir cat iBody 2** produced a three-fold higher total number of significantly enriched proteins compared to **HRP iBody 1** and a nine-fold increase relative to **RFT iBody 3**. These findings were unexpected considering the highly selective spatiotemporal protein labeling previously reported for µMap, which employs short-lived carbenes as the reactive biotin-carrying intermediates with a diffusion radius of 2–4 nm^15^. However, Knutson et al. also observed a decrease in actual µMap labeling precision compared to theoretical predictions^30^. Using a super-resolution microscopy-based workflow, they measured biotinylation clusters with a radial size of approximately 50 nm. They attributed this reduced precision to the size of the complex comprising the primary targeting antibody and Ir-conjugated secondary antibody (~ 15 nm per antibody). However, as the **Ir cat iBody 2** polymer conjugate is about half the molecular weight of a single antibody, its size is unlikely to significantly decrease labeling precision. The higher reactivity of carbenes, which readily cross-link with X–H bonds in biomolecules, compared to phenoxyl radicals generated by HRP and RFT that primarily target less abundant tyrosine residues, may also play a role.

### 45 common proteins associated with the GCPII microenvironment were identified by all three labeling techniques

**Fig. 3:**
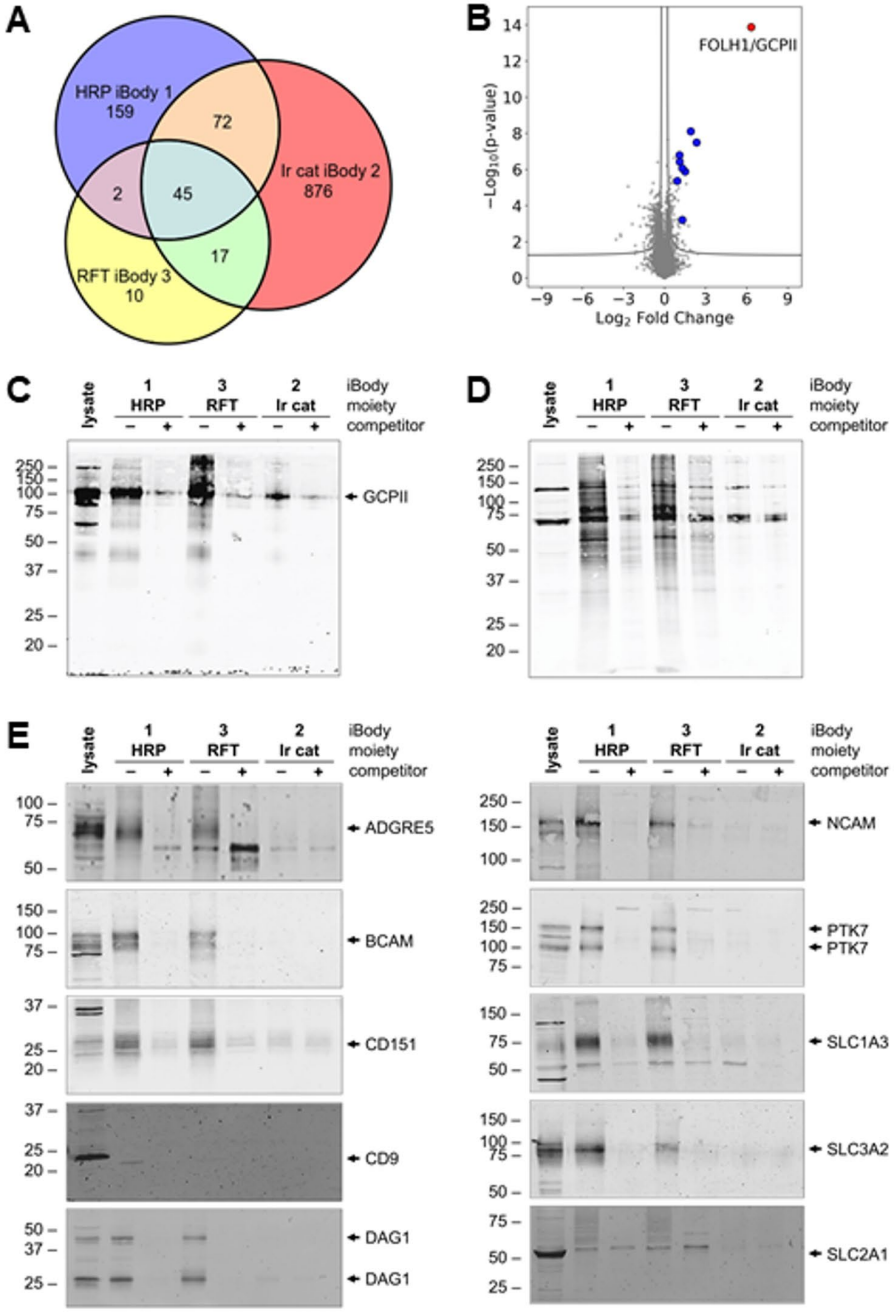
GCPII microenvironment analysis. (**A**) Venn diagram analysis of highly enriched proteins reveals a core overlap of 45 proteins identified by targeted **HRP iBody 1**, **Ir cat iBody 2**, and **RFT iBody 3**-based PPL, along with an additional 91 proteins detected by any pair of methods. (**B**) Pulldown of GCPII and proximal proteins from U251 MG–GCPII lysate (without prior biotinylation) using anti-GCPII **iBody 5** (xGCPII and biotin). **iBody 6** (biotin alone) was used in control experiments. The quantitative proteomics volcano plot displays significantly enriched proteins (*P* < 0.05, FDR-corrected, *n* = 5 replicates). GCPII (highlighted in red) emerged as the most enriched protein. Eight proteins exhibiting > 2-fold enrichment and identified previously by at least two biotinylation strategies are marked in blue (CD81, CD9, GPM6A, ITGB1, MYADM, PROCR, PTGFRN, and THY1). (**C-E**) Western blot analyses of proximity biotinylation on the U251 MG-GCPII cell surface using **HRP**, **Ir cat**, and **RFT**-conjugated iBodies. The GCPII specific inhibitor 2-PMPA was utilized as a competitor in control reactions, eluates from corresponding experimental replicates were pooled for analysis. SDS-PAGE of enriched proteins followed by immunostaining with anti-GCPII antibody (**C**), streptavidin (**D**), and antibodies against ADGRE5, BCAM, CD151, DAG1, NCAM1, PTK7, SLC1A3, and SLC3A2 (**E**) shows strong biotinylation by **HRP** and **RFT**-conjugated iBodies. CD9 was efficiently biotinylated exclusively with HRP iBody 1, enabling its identification on the Western blot. In contrast, SLC2A1 was insufficiently biotinylated by all of the iBodies tested, preventing its detection on the Western blot. **Ir cat iBody 2** exhibited less efficient biotinylation, resulting in no observable signal of proteins other than GCPII. Streptavidin immunostaining (**D**) revealed the same overall pattern of protein biotinylation: robust labeling by **HRP**- and **RFT**-conjugated iBodies and less effective labeling by **Ir cat iBody 2**. Uncropped Western blot images are shown in Supplementary Fig. S4 (anti-GCPII and streptavidin immunostaining) and Fig. S5 (immunostaining of identified proteins).

Although our experiments were performed on transfected cells, the approach offers a reliable platform for analysis of significantly enriched proteins to investigate the proximal interactome of GCPII in other contexts. A total of 45 common proteins met the enrichment criteria (≥ 4-fold enrichment, FDR < 0.01) across all three labeling techniques (Table 1), with an additional 91 proteins identified by any pair of methods (Fig. 3A, Supplementary Table S2). The first group of proteins comprises intrinsic membrane components. A small subset of proteins in the second group is intracellular (marked with an asterisk in Supplementary Table S2). Intracellular labeling may result from incomplete inhibition of GCPII internalization following iBody binding, as seen by confocal microscopy (Supplementary Fig. S1). Both biotinylation reagents can cross the cell membrane to a limited extent^31,32^.

**Table 1:** Proteins identified across the three biotinylating techniques using **HRP iBody 1**, **Ir Cat iBody 2**, and **RFT iBody 3**.

Gene	Protein
ADGRE5 $	Adhesion G protein-coupled receptor E5
ALCAM	CD166 antigen
ATP1A1	Sodium/potassium-transporting ATPase subunit alpha-1
BCAM $	Basal cell adhesion molecule
CADM1	Cell adhesion molecule 1
CD109	CD109 antigen
CD151 $	CD151 antigen
CD44	CD44 antigen
CD58	Lymphocyte function-associated antigen 3
CD70	CD70 antigen
CD81 ↓	CD81 antigen
CD9 $↓	CD9 antigen
CDH13	Cadherin-13
CDH2	Cadherin-2
CDH4	Cadherin-4
DAG1 $	Dystroglycan 1
DCBLD2	Discoidin, CUB and LCCL domain-containing protein 2
DDR2	Discoidin domain-containing receptor 2
EPHA2	Ephrin type-A receptor 2
FOLH1 $↓	Glutamate carboxypeptidase 2 (GCPII)
GNA11	Guanine nucleotide-binding protein subunit alpha-11
GNAI2	Guanine nucleotide-binding protein G(i) subunit alpha-2
GPM6A ↓	Neuronal membrane glycoprotein M6-a
HLA-A	HLA class I histocompatibility antigen, A alpha chain
IL1RAP	Interleukin-1 receptor accessory protein
ITGA3	Integrin alpha-3
ITGAV	Integrin alpha-V
ITGB1 ↓	Integrin beta-1
ITGB8	Integrin beta-8
MMP14	Matrix metalloproteinase-14
MOXD1	DBH-like monooxygenase protein 1
MRC2	C-type mannose receptor 2
NRP2	Neuropilin-2
PTGFRN ↓	Prostaglandin F2 receptor negative regulator
PTK7 $	Inactive tyrosine-protein kinase 7
SLC16A1	Monocarboxylate transporter 1
SLC16A3	Monocarboxylate transporter 4
SLC1A5	Amino acid transporter
SLC2A1	Solute carrier family 2, facilitated glucose transporter member 1
SLC3A2 $	Amino acid transporter heavy chain SLC3A2
SLC44A2	Choline transporter-like protein 2
SLC7A5	Large neutral amino acids transporter small subunit 1
TFRC	Transferring receptor protein 1
THY1 ↓	Thy-1 membrane glycoprotein
TPBG	Trophoblast glycoprotein

$ Proteins validated by Western blots.

↓ Proteins validated by pulldown of GCPII using U251 MG-GCPII lysate.

### Eight identified proteins were validated by a GCPII pulldown using cell lysate

To validate our findings, we performed a GCPII pulldown from U251 MG-GCPII cell lysate using **iBody 5** (xGCPII, biotin) without prior biotinylation. **iBody 6**, which contains biotin but not xGCPII, was used as a background binding negative control. MS analysis identified GCPII as the most enriched protein, along with eight additional proteins previously detected in proximity labeling experiments: CD81 (CD81 antigen), CD9 (CD9 antigen), GPM6A (neuronal membrane glycoprotein M6-a), ITGB1 (integrin β1), MYADM (myeloid-associated differentiation marker), PROCR (endothelial protein C receptor), PTGFRN (prostaglandin F2 receptor negative regulator), and THY1 (Thy-1 membrane glycoprotein) (Fig. 3B).

### Western blot analyses validated GCPII and nine additional identified proteins

Finally, we confirmed the presence of selected hit proteins in the enriched pool using Western blotting. Targeted **HRP** and **RFT** labeling produced robust biotinylation and subsequent enrichment of GCPII (Fig. 3C), along with eight additional proteins: ADGRE5 (adhesion G protein-coupled receptor E5), BCAM (basal cell adhesion molecule), CD151 (CD151 antigen), DAG1 (dystroglycan 1), NCAM1 (neural cell adhesion molecule 1), PTK7 (inactive tyrosine-protein kinase 7), SLC1A3 (excitatory amino acid transporter), and SLC3A2 (amino acid transporter heavy chain). CD9 antigen was efficiently biotinylated, appearing on the Western blot, exclusively with **HRP iBody 1.** In contrast, SLC2A1 (GLUT1) was not sufficiently biotinylated to allow its detection on the Western blot by any of the iBodies tested. **Ir cat iBody 2** exhibited lower GCPII biotinylation, with no other detectable proteins on the blots (Fig. 3C, E). The same pattern of overall protein biotinylation was also evident with streptavidin immunostaining: strong labeling by **HRP**- and **RFT**-conjugated iBodies and less effective labeling by **Ir cat iBody 2** (Fig. 3D).

### Multiple identified proteins participate in glutamate and folate metabolism and are also implicated in cancer development

Several proteins identified by our PPL techniques in the microenvironment of GCPII may contribute to the enzyme’s roles in glutamate and folate metabolism, as well as in cancer progression.

GCPII plays a direct role in folate metabolism and transport by hydrolyzing polyglutamated folates, thereby enhancing their bioavailability. CD320, the receptor for transcobalamin-bound vitamin B12, is essential for one-carbon metabolism, which is closely linked to folate metabolism^33^. Several solute carrier (SLC) transporters also participate in these metabolic pathways. SLC1A5 (ASCT2) and SLC7A5 (LAT1) mediate glutamine uptake, a key component of cancer metabolism^34,35^. SLC3A2 (4F2hc, CD98) forms a heterodimer with LAT1 and acts as a chaperone, promoting light chain LAT1 subunit recruitment to the plasma membrane, thereby enhancing amino acid transport, supporting rapid cell proliferation, and contributing to prostate carcinoma progression^36^. SLC2A1 (GLUT1), frequently upregulated in cancer, enables increased glucose uptake for glycolysis.

Several of the proteins we identified mediate cell-cell adhesion, a process crucial for maintaining tissue architecture and regulating cell migration. These include ADGRE5 (CD97), certain cadherins (CDH2, CDH4, and CDH13), CADM1 (cell adhesion molecule 1), CD9, CD81, CD151, DAG1 (dystroglycan), EPHA2 (ephrin type-A receptor 2), integrins, LFA-3 (CD58), and THY1 (Thy-1 membrane glycoprotein). Dysregulation of these proteins in cancer can lead to reduced cell adhesion, increased migration, and resistance to apoptosis^37–40^. For example, CD97 can contribute to the invasion of prostate cancer cells^41^. ITGAV (integrin αV) and ITGB8 (integrin β8) are key regulators of extracellular matrix (ECM) remodeling and TGF-β activation^42^. DCBLD2 (discoidin, CUB and LCCL domain-containing protein 2) and MMP14 (matrix metalloproteinase-14) are also implicated in tumor invasiveness, either via epithelial-mesenchymal transition signaling or through degradation of ECM components^43,44^.

DCBLD2 reportedly interacts with ITGB1 (integrin β1)^43^, a key component of the focal adhesion pathway^45^. Several studies have elucidated the relationship between ITGB1 and GCPII. Conway et al. observed that GCPII modulates ITGB1 signaling and p21-activated kinase 1 (PAK-1) activity, both of which are critical for endothelial cell invasion and angiogenesis^46^. Caromile et al. found that increased GCPII expression in prostate tumors promotes cancer progression by disrupting ITGB1 and IGF-1R complex signaling within the MAPK pathway, thereby facilitating AKT pathway activation^47^. Additionally, Perico et al. reported that GCPII forms a functionally active macromolecular complex with ITGB1, filamin A, p130CAS, c-Src, and EGFR at LNCaP or PC3-PSMA cell membranes, a process that is essential for GCPII-dependent cell growth and survival in 3D culture^48^. Notably, ITGB1 was among the most enriched proteins identified across our targeted PPL experiments. EGFR (epidermal growth factor receptor), the other constituent of the complex, and ITGA3 (integrin α3) were both detected in this study. Furthermore, ITGA3 has been identified by Oakley J. V. et al. as a key EGFR interactor, based on A549 cell–surface labeling of the EGFR interactome^20^.

GCPII, along with several associated proteins, has been implicated in cancer progression^49–51^. EGFR is frequently dysregulated in cancer^52^ and is involved in glutamate signaling^53,54^. CD44 contributes to tumor progression by interacting with hyaluronic acid, osteopontin, and MMPs, thereby enhancing tumor cell proliferation, migration, and invasion^55^. CD109 may advance carcinoma progression via EGFR-mediated STAT3 regulation, impacting tumorigenicity and aggressiveness, as shown for cervical squamous carcinoma^56^. TFRC (transferrin receptor 1) supports iron uptake, which is crucial for the rapid proliferation of cancer cells^57^.

Several of the proteins identified in our study are involved in immune regulation. Major histocompatibility complex class I molecules HLA-A and HLA-B are central to immune recognition, mediating T-cell activation against cancer cells. Certain HLA genetic variants are associated with an increased risk of developing prostate cancer^58^. PTK7 and TPBG (trophoblast glycoprotein) have been implicated in Wnt/β-catenin signaling, further contributing to adhesion and immune evasion^59^. Neural cell adhesion molecules ALCAM and GPM6A play roles in neural development and cancer cell migration^60,61^.

In the future, we plan to apply the developed biotinylating iBodies platform for studies of other membrane protein targets on primary cell lines with limited availability. Therefore, we aimed to determine whether it is possible to reduce the number of experimental replicates while maintaining statistical significance. We generated volcano plots using PPL data either from all of the performed experiments (hexaplicates) or from triplicates (created from hexaplicates by random selection). The resulting volcano plots indicated that triplicate analysis yields a well-defined subset of proteins above the FDR threshold for **Ir cat** and **RFT** labeling experiments. In contrast, **HRP** labeling required a higher number of replicates to achieve comparable statistical confidence (Supplementary Fig. S6).

### Summary

Our novel iBody proximity labeling platform offers several key benefits advantageous for elucidating the spatial interactomes of proteins of interest or identifying membrane protein targets of biologically active molecules. HPMA polymers provide a soluble and hydrophilic framework with minimal nonspecific binding and prevent penetration through the lipid bilayer membrane. The incorporation of multiple targeting ligands and biotinylating moieties within the scaffold can enhance target binding through the avidity effect, while also augmenting labeling. Among the three methods tested, the highly precise **RFT**-based iBody labeling approach demonstrates the greatest potential due to its efficient light-triggered activation and minimal background.

The set of proteins identified in proximity to GCPII reveals a complex network that can help enhance cancer cell metabolism, migration, invasiveness, and progression, while also playing a role in modulating immune evasion. Notably, two of these proteins, ITGB1 and EGFR, were previously reported to form a macromolecular complex with GCPII, validating our findings and supporting the relevance of the identified interactome.

## Supplementary Information

Below is the link to the electronic supplementary material.

Supplementary Material 1

Supplementary Material 2

Supplementary Material 3

Supplementary Material 4

## Data Availability

The mass spectrometry proteomics data have been deposited to the ProteomeXchange Consortium via the PRIDE partner repository with the dataset identifier PXD068378.
