# Supplementary material for: Uncovering the glutamate carboxypeptidase II microenvironment using a multi-labeling proteomic approach
Source: Sci Rep. 2025 Nov 27;15:45461. doi: 10.1038/s41598-025-28826-5 (PMC12749419; doi:10.1038/s41598-025-28826-5)
Supplement: Supplementary file 4 — Supplementary Material 4 [file 41598_2025_28826_MOESM4_ESM.docx]

**Uncovering the Glutamate Carboxypeptidase II Microenvironment Using a Multi-labeling Proteomic Approach**

**Supplementary Information**

Jana Pokorná, ^[a]^ Martin Hadzima, ^[a]^ Alena Křenková, ^[a]^ Joshua D. Smith, ^[a]^ Vladimír Šubr, ^[b]^ Robin Kryštůfek, ^[a]^ Martin Hubálek, ^[a]^ Jana Starková, ^[a]^ Karolína Šrámková, ^[a]^ Tomáš Etrych, ^[b]^ Libor Kostka, ^[b]^ František Sedlák, ^[a, c]^ Jan Konvalinka, ^[a]^ * Pavel Šácha^[a]^ *

*[a] Jana Pokorná, Martin Hadzima, Alena Křenková, Joshua D. Smith, Robin Kryštůfek, Martin Hubálek, Jana Starková, Karolína Šrámková, František Sedlák, Jan Konvalinka, Pavel Šácha*

*Institute of Organic Chemistry and Biochemistry*

*Czech Academy of Sciences*

*Flemingovo nám 542/2, 160 00, Prague, Czech Republic*

* *Correspondence: jan.konvalinka@uochb.cas.cz*

*pavel.sacha@uochb.cas.cz*

*[b] Vladimír Šubr, Tomáš Etrych, Libor Kostka,*

*Department of Biomedical Polymers*

*Institute of Macromolecular Chemistry*

*Czech Academy of Sciences*

*Heyrovského nám 2, 160 00, Prague, Czech Republic*

*[c] František Sedlák*

*Department of Biochemistry and Experimental Oncology*

*First Faculty of Medicine*

*Charles University*

*Kateřinská 32, 121 08, Prague, Czech Republic*

*TABLE OF CONTENTS*

***Supplementary Tables* ....................................................................................................................3**

**Table S1: Composition and basic characteristics of the iBodies.............................................3**

**Table S2: Proteins identified by any pair of biotinylating techniques....................................3
*Supplementary Figures*** ***.* .................................................................................................................6
 Fig. S1: Prevention of GCPII internalization visualized by confocal microscopy**  **................6
 Fig. S2: Photonic control over the GCPII labeling reaction with RFT iBody 3.....................7
 Fig. S3: Irradiation chambers used for photocatalyzed labeling in 28 mm scintillation vials**

**(IOCB Development workshop).................................................................................................7**

**Fig. S4: Western blot analyses (uncropped images) of proximity biotinylation on the U251**

**MG-GCPII cell surface using HRP, Ir cat, and RFT-conjugated iBodies; anti-GCPII and**

**streptavidin immunostaining…………………………………………………………………..8
 Fig. S5: Western blot analyses (uncropped images) of proximity biotinylation on the U251**

**MG-GCPII cell surface using HRP, Ir cat, and RFT-conjugated iBodies; immunostaining**

**of identified proteins.…….…..................................................................................................…9**

**Fig. S6: PPL of GCPII on live U251 MG–GCPII cells using targeted iBodies: comparing**

**hexaplicates and triplicates.......................................................................................................10
*Supplementary methods.*...............................................................................................................11**

**Synthesis of low-molecular-weight compounds** **......................................................................11**

**Biotinyl-PEG-5-diazirine (Compound 1)................................................................................11**

**Riboflavin tetraacetate (Compound 2)....................................................................................12**

**Riboflavin tetraacetate-C_6_-NHBoc (Compound 3)................................................................12**

**Riboflavin tetraacetate-C_6_-NH_2_ (RFT)....................................................................................13**

**2-(4'-(2-methoxypropan-2-yl)-[2,2'-bipyridin]-4-yl)propan-2-ol (Compound 4) )..............13**

**4-(20-azido-2-methyl-3,6,9,12,15,18-hexaoxaicosan-2-yl)-4'-(2-methoxypropan-2-yl)-2,2'-**

**bipyridine (Compound 5)**  **.........................................................................................................14**

**Compound** **6 ...............................................................................................................................14**

**N_3_-PEG_5_-Ir-catalyst (Ir cat)….………………..…………………...........................................15**

**Synthesis of monomers, polymer precursors, and polymer conjugates (iBodies)...............15**

**Materials ..................................................................................................................................15**

**Synthesis of monomers and chain transfer agents)..............................................................16**

**Preparation of azide-modified HRP......................................................................................17**

**Synthesis of polymer precursors............................................................................................17**

**Synthesis of iBodies.................................................................................................................17**

**Synthesis of HRP iBody 1.......................................................................................................17**

**Synthesis of Ir cat iBody 2......................................................................................................18**

**Synthesis of RFT iBody 3........................................................................................................19**

**Synthesis of iBody 4................................................................................................................20**

**Synthesis of iBody 5................................................................................................................20**

**Synthesis of iBody 6................................................................................................................21**

**Characterization of polymer precursors and iBodies..........................................................22**

**Confocal microscopy..................................................................................................................22**

**Proximity-selective labeling on live U-251 MG–GCPII cells for Western blot and quantitative LC-MS/MS analyses............................................................................................23**

**Proximity-selective HRP iBody 1 labeling and untargeted free HRP labeling.................23**

**Proximity-selective Ir cat iBody 2 labeling and untargeted free Ir cat labeling...............24**

**Proximity-selective RFT iBody 3 labeling and untargeted free RFT labeling..................25**

**Liquid chromatography-tandem mass spectrometry (LC-MS/MS) data acquisition.........26**

**Protein identification and quantification..............................................................................27**

**SDS-PAGE and Western blotting............................................................................................28**

**Pull-down/immunoprecipitation of GCPII and adjacent proteins from U-251 MG–GCPII lysate ...........................................................................................................................................29**

**References.....................................................................................................................................29**

***Supplementary Tables***

Supplementary Table S1. **Composition and basic characteristics of the iBodies**

|  | **iBody 1** | **iBody 2** | **iBody 3** | **iBody 4** | **iBody 5** | **iBody 6** |
| --- | --- | --- | --- | --- | --- | --- |
| ***M_w_* [kg.mol^-1^]*** | 67.0 | 66.0 | 62.0 | 72.0 | 26.6 | 78.5 |
| ***M_n_* [kg.mol^-1^]*** | 59.8 | 57.0 | 59.0 | 60.0 | 24.8 | 66.0 |
| ***Ð* *** | 1.12 | 1.16 | 1.05 | 1.2 | 1.07 | 1.19 |
| ***M_w_***’ **[kg.mol^-1^]*** | 319 | 79.2 | 79.5 | 87.7 | 30.9 | 81.3 |
| **No. of HRP moieties** | 5.5 | - | - | - | - | - |
| **No. of Ir cat moieties** | - | 6.3 | - | - | - | - |
| **No. of RFT moieties** | - | - | 15 | - | - | - |
| **No. of GCPII inhibitor moieties** | 8.4 | 11 | 11 | 15 | 4.6 | - |
| **No. of** **biotin units** | - | - | - | - | 2.7 | 9.8 |
| **No. of ATTO 488 units** | - | - | - | 6.1 | - | - |

* *M_w_*, weight-average molecular weights of the precursor; *M_n_*, number-average molecular weights of the precursor; *Ð*, dispersity of the precursor; *M_w_*’, *M_w_* of polymer precursor, enlarged by the sum of apparent molecular weights of all conjugated ligands; No. per polymer, an average number of conjugated ligands per molecule of HPMA polymer scaffold.^1^

Supplementary Table S2. **Proteins identified by any pair of biotinylating techniques**

| **Proteins identified by couples of PPL methods** | | |
| --- | --- | --- |
| **HRP ∩ Ir cat** | **HRP ∩ RFT** | **Ir cat ∩ RFT** |
| ABCC4 | NCAM1 $ | ACSF3 ✼ |
| ADAM10 | PODXL | CAMK2G |
| ADAM9 |  | CD320 |
| ATP11C |  | CLPX ✼ |
| ATP1B1 |  | EDC4 ✼ |
| ATP1B3 |  | FARS2 ✼ |
| ATP2B1 |  | HLA-B |
| ATP2B4 |  | MOGS |
| BSG |  | MYO1C |
| BTAF1 ✼ |  | OXNAD1 ✼ |
| CAV1 |  | PELO ✼ |
| CD47 |  | PPP6R3 ✼ |
| CD59 |  | RRAS2 |
| CEMIP2 |  | SLFN5 ✼ |
| CHD4 |  | TUFM ✼ |
| CKLF |  | VWA8 ✼ |
| CTNNA1 |  | WARS2 ✼ |
| CXADR |  |  |
| CYBRD1 |  |  |
| DDX21 ✼ |  |  |
| DNAJC5 |  |  |
| EGFR |  |  |
| EMP3 |  |  |
| EPHA3 |  |  |
| EVA1A |  |  |
| GGCX |  |  |
| GLIPR2 |  |  |
| GNA13 |  |  |
| GNAI3 |  |  |
| GNAS |  |  |
| GNG12 |  |  |
| GPM6B |  |  |
| ITGA5 |  |  |
| LGALS1 ✼ |  |  |
| LIMCH1 |  |  |
| LNPEP |  |  |
| LRRC8A |  |  |
| MYADM ↓ |  |  |
| MYBBP1A ✼ |  |  |
| NECTIN2 |  |  |
| NPTN |  |  |
| NUMA1 |  |  |
| NUP35 ✼ |  |  |
| PLP2 |  |  |
| PLPP2 |  |  |
| PROCR ↓ |  |  |
| PTPRJ |  |  |
| SCAMP4 |  |  |
| SERINC5 |  |  |
| SLC12A2 |  |  |
| SLC16A2 |  |  |
| SLC1A3 $ |  |  |
| SLC1A4 |  |  |
| SLC29A1 |  |  |
| SLC38A1 |  |  |
| SLC39A10 |  |  |
| SLC39A14 |  |  |
| SLC44A1 |  |  |
| SLC4A7 |  |  |
| SLC5A3 |  |  |
| SLC6A6 |  |  |
| SLC7A1 |  |  |
| SLC7A11 |  |  |
| SNAP23 |  |  |
| SORT1 |  |  |
| STEAP3 |  |  |
| STXBP3 |  |  |
| SV2A |  |  |
| SYMPK |  |  |
| TM9SF2 |  |  |
| TMEM30A |  |  |
| TSPAN9 |  |  |

✼ Nonmembrane protein localization

$ Proteins validated by Western blots

↓ Proteins validated by pulldown of GCPII using U251 MG-GCPII lysate

***Supplementary Figures***


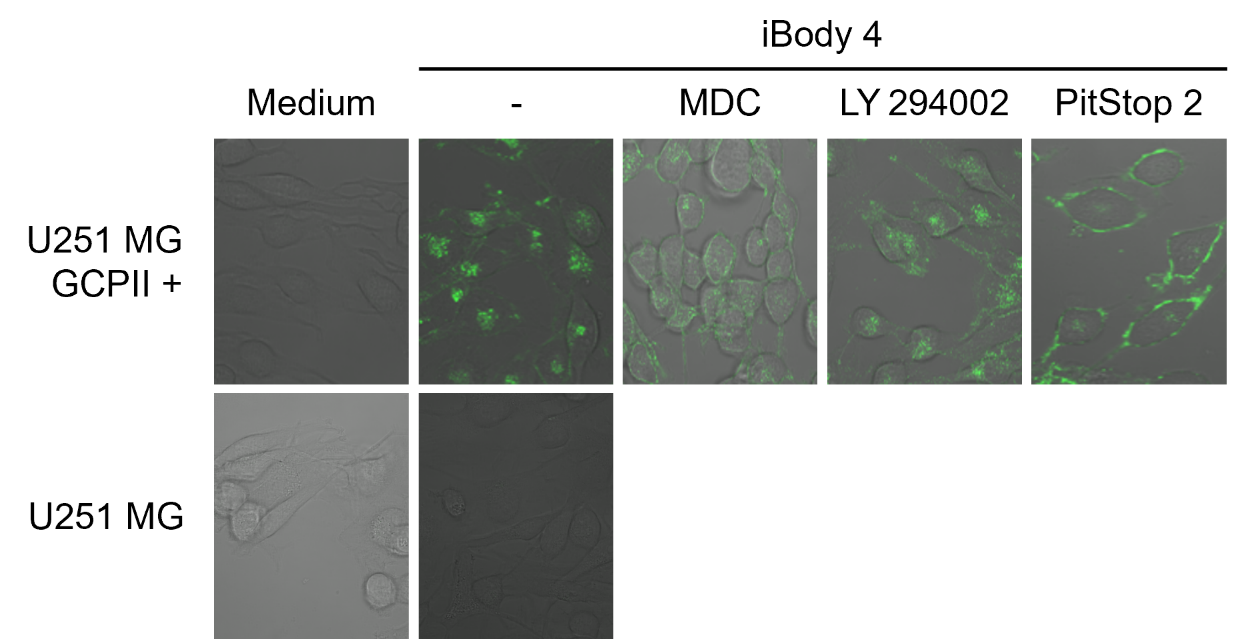


Supplementary Fig. S1. **Prevention of GCPII internalization visualized by confocal microscopy.** Cells positive (U251 MG–GCPII) and negative (U251 MG) for GCPII expression were stained with **iBody 4** **(**GCPII inhibitor, ATTO 488). The internalization inhibitors MDC (monodansyl cadaverine), LY 294002, and PitStop 2 were compared. Following a 1-hour incubation with **iBody 4,** PitStop 2 effectively prevented internalization, whereas MDC and LY 294002 did not.


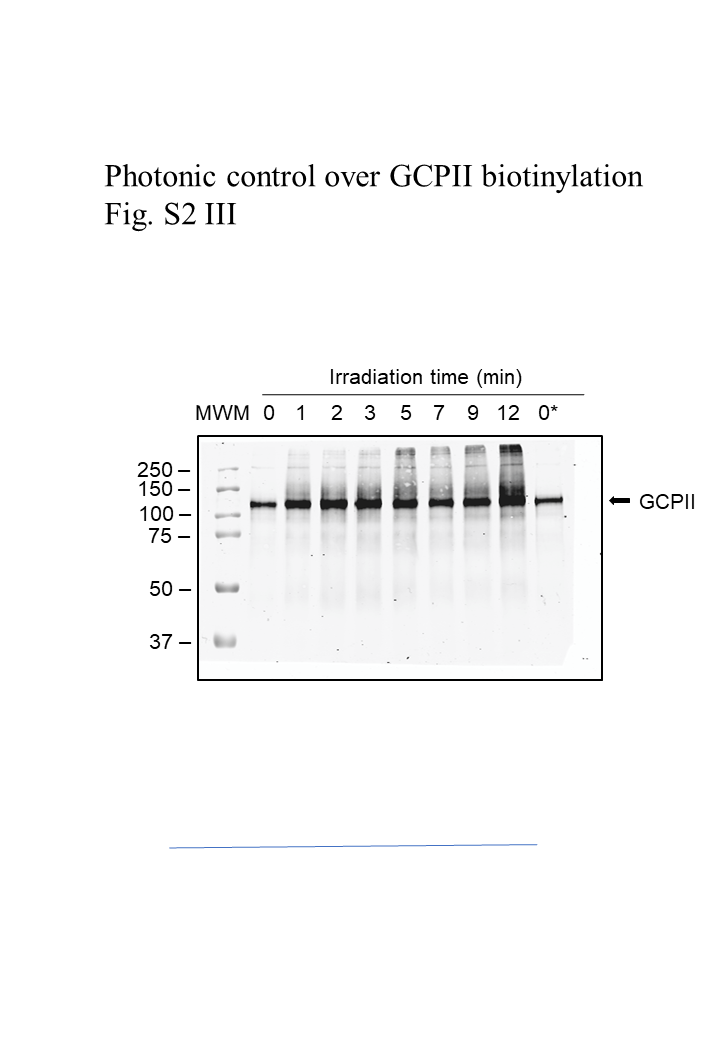


Supplementary Fig. S2. **Photonic control over the GCPII labeling reaction with** RFT iBody 3**.** U251 MG–GCPII cells were irradiated with 400 nm light in the presence of **RFT iBody 3** and biotinyl-tyramide. Aliquots for analysis were taken after 1, 2, 3, 5, 7, 9, and 12 minutes. After labeling, cells were lysed and labeled proteins were enriched via streptavidin-bead pulldown. Enriched GCPII was analyzed by the Western blot using the anti-GCPII monoclonal antibody GCP-04 and IRDye 800CW goat anti-mouse secondary antibody. Image was acquired using Odyssey CLx® Imaging System (LI-COR®), **and contrast was adjusted to the minimum setting.** GCPII biotinylation increased with irradiation time, reaching a plateau at 5 minutes. No labeling was observed during biotinyl-tyramide incubation in the absence of light (0 min lane), even with prolonged incubation of 12 minutes with biotinyl-tyramide (0* min lane).


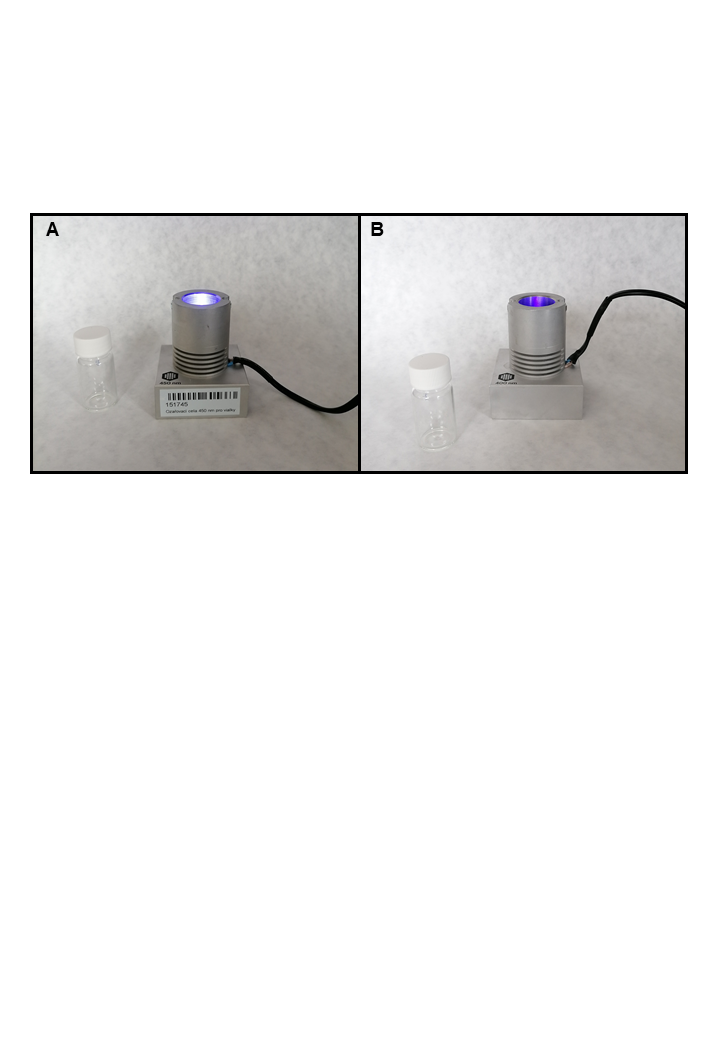


Supplementary Fig. S3. **Irradiation chambers used for photocatalyzed labeling in 28 mm scintillation vials** **(IOCB Development workshop).** (A) Chamber for 450 nm irradiation used for Ir catalyst labeling (OSRAM LZ1-00DB00 LED, power rating 3.6 W, radiant flux 1.1 W). (B) Chamber for 400 nm irradiation used for RFT labeling (OSRAM LZ1-00UB0R-00U6 LED, power rating 3.5 W, radiant flux 1.1 W)


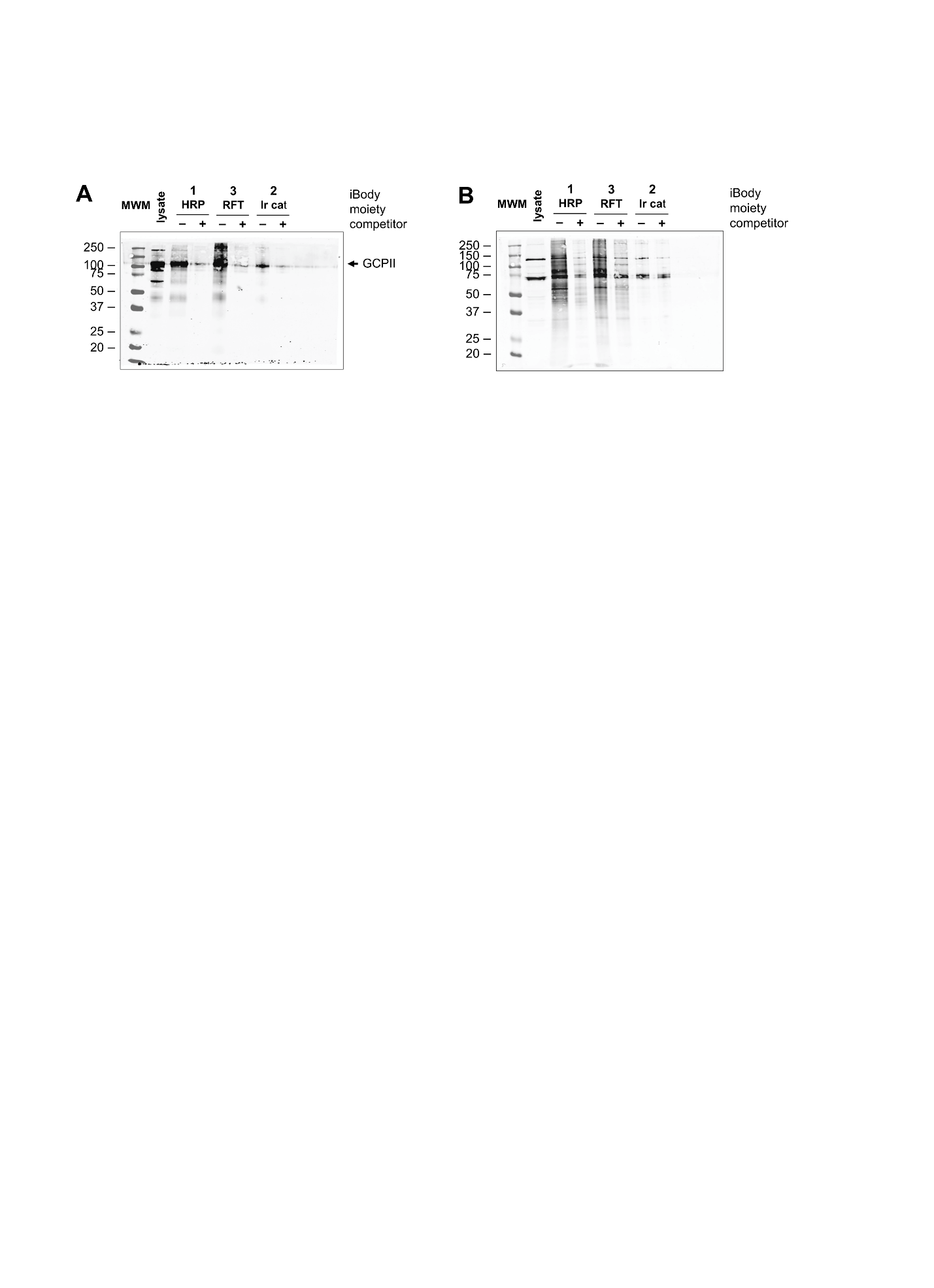


Supplementary Fig. S4. Western blot analyses (uncropped images) of proximity biotinylation on the U251 MG-GCPII cell surface using **HRP**, **Ir cat**, and **RFT**-conjugated iBodies; anti-GCPII and streptavidin immunostaining. SDS-PAGE of enriched proteins followed by immunostaining with anti-GCPII antibody (A) and streptavidin (B). The GCPII-specific inhibitor 2-PMPA was used as a competitor in control reactions.


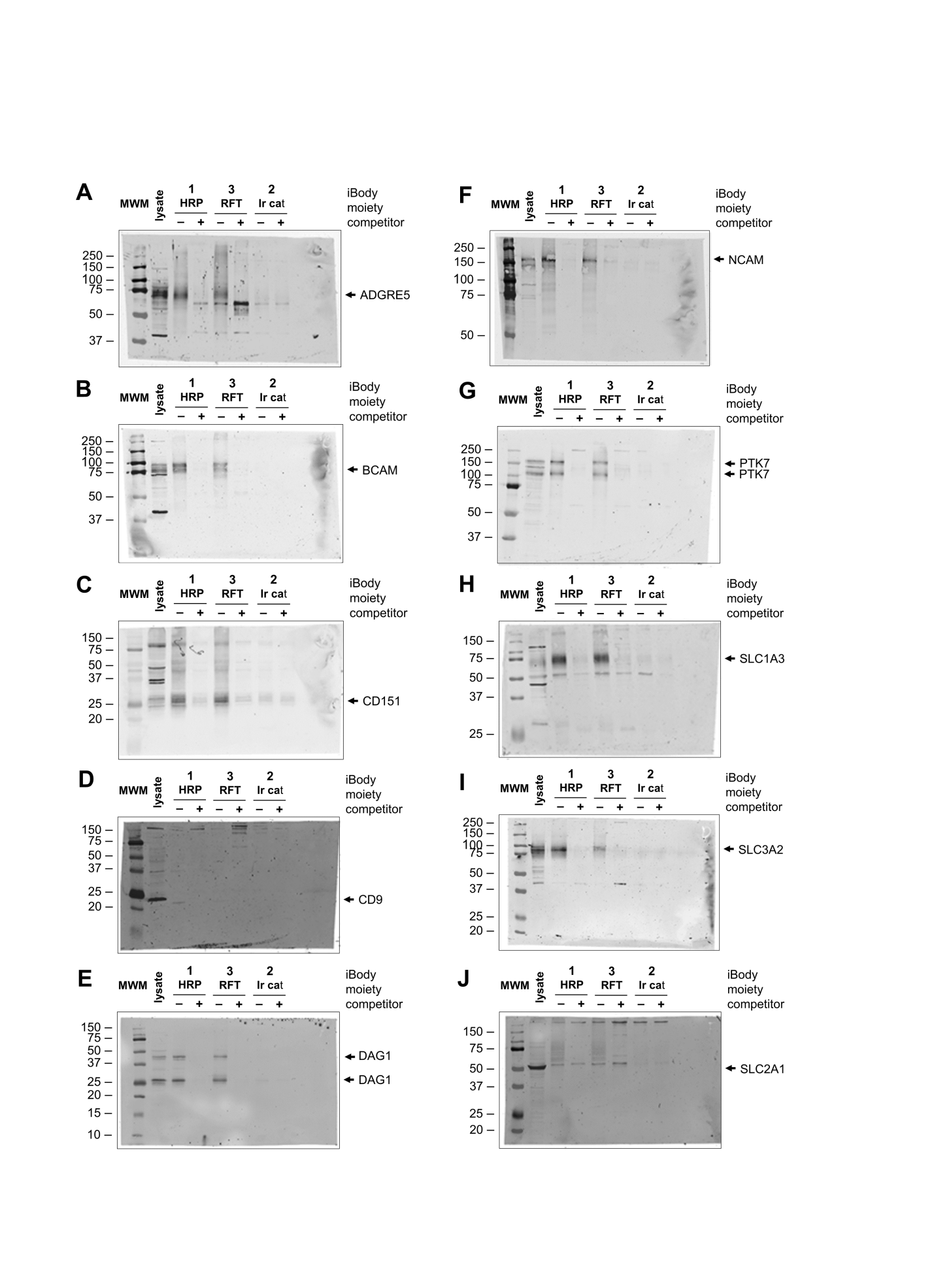


Supplementary Fig. S5 . Western blot analyses (uncropped images) of proximity biotinylation on the U251 MG-GCPII cell surface using **HRP**, **Ir cat**, and **RFT**-conjugated iBodies; immunostaining of identified proteins. SDS-PAGE of enriched proteins was followed by immunostaining with antibodies against ADGRE5 (A), BCAM (B), CD151 (C), CD9 (D), DAG1 (E), NCAM (F), PTK7 (G), SLC1A3 (H), SLC3A2 (I), and SLC2A1 (J). The GCPII-specific inhibitor 2-PMPA was used as a competitor in control reactions.


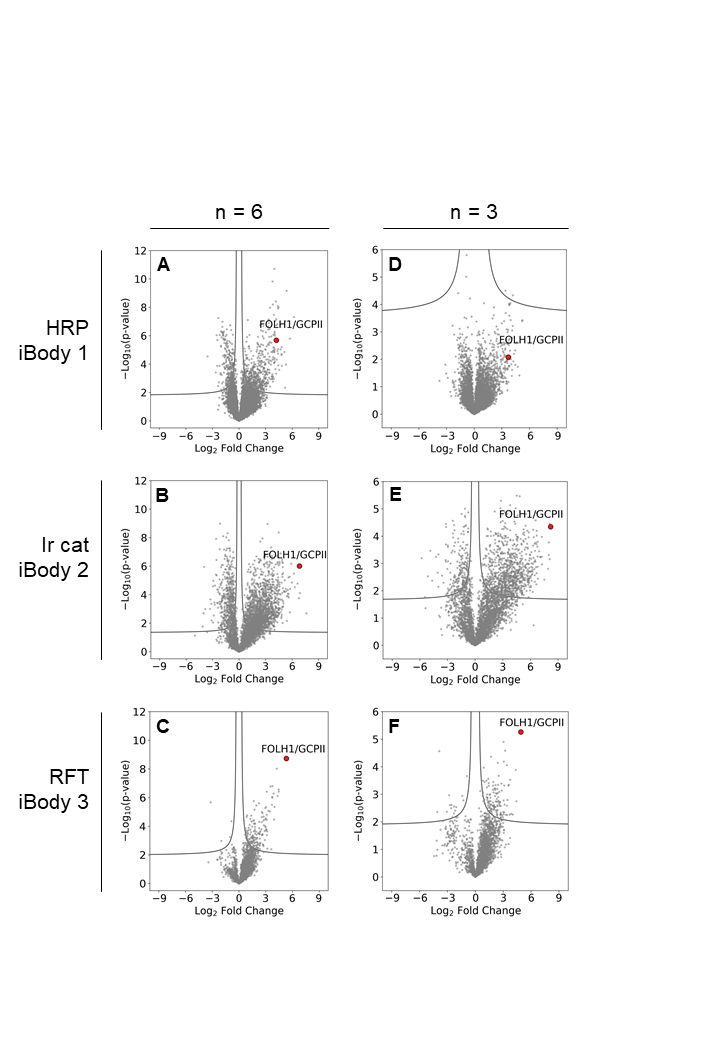


Supplementary Fig. S6. **PPL of GCPII on live U251 MG–GCPII cells using targeted iBodies: comparing hexaplicates and triplicates.** Quantitative proteomics volcano plots highlighting significantly enriched proteins (false discovery rate [FDR]–corrected *P* < 0.01). Averaged log₂ enrichment ratios [GCPII-targeted biotinylation by **HRP iBody 1** (A, D), **Ir cat iBody 2** (B, E), and **RFT** **iBody 3** (C, F) vs. reactions in the presence of the competitive inhibitor 2-PMPA] on the x-axis and –log₁₀ *P* values on the y-axis are depicted. (A–C) *n* = 6 replicates, (D–F) *n* = 3 replicates. GCPII is highlighted in red.

Volcano plots suggest that targeted **Ir cat** and **RFT** labeling may require fewer replicates, as analysis of triplicates identifies proteins above the FDR threshold, whereas **HRP** labeling requires more replicates for similar confidence.

***Supplementary methods***

**Synthesis of low-molecular-weight compounds**

**Biotinyl-PEG-5-diazirine (**Compound **1)**

Biotin (269 mg, 1.1 mmol, 1.1 eq.) was dissolved in dry DMF (3 mL). DIPEA (0.87 mL, 5.0 mmol, 5.0 eq.) was added, followed by TSTU (301 mg, 1.0 mmol, 1.0 eq.). The reaction mixture was stirred for 1 hour at room temperature before NH_2_-PEG-6-NHBoc (342 mg, 0.9 mmol, 0.9 eq.) in dry DMF (1 mL) was added. The reaction mixture was stirred overnight at room temperature. The product was purified by reversed-phase flash chromatography (C18 Aqua, gradient 0% → 100% MeCN in H_2_O + 0.1% TFA) to yield biotin-NH-PEG-6-NHBoc (448 mg, 82%) as an off-white lyophilizate.

Biotin-NH-PEG-6-NHBoc (225 mg, 0.37 mmol, 1.0 eq.) was dissolved in a 1:1 mixture of TFA/DCM (2 mL). The reaction mixture was stirred for 3 hours at room temperature and the volatiles were removed. The residue was dried in a high vacuum overnight to yield biotin-NH-PEG-6-NH_2_ (188 mg, quant.).

4-(3-Trifluoromethyl)-3H-diazirin-3-yl)benzoic acid (104 mg, 0.45 mmol, 1.1 eq.) was dissolved in dry DMF (1 mL). DIPEA (0.36 mL, 2.1 mmol, 5.0 eq.) was added, followed by TSTU (124 mg, 0.41 mmol, 1.0 eq.). The reaction mixture was stirred for 1 hour at room temperature before biotin-NH-PEG-6-NH_2_ (188 mg, 0.37 mmol, 0.9 eq.) in dry DMF (2 mL) was added. The reaction mixture was stirred overnight at room temperature. The product was purified by reversed-phase flash chromatography (C18 Aqua, gradient 0% → 100% MeCN in H_2_O + 0.1% TFA) to yield biotinyl-diazirine **(1)** (173 mg, 65%) as a white lyophilizate.

**^1^H NMR** (400 MHz, CD_3_OD) δ 7.95 – 7.90 (m, 2H), 7.38 – 7.31 (m, 2H), 4.49 (ddd, *J* = 7.9, 5.0, 0.9 Hz, 1H), 4.30 (dd, *J* = 7.9, 4.5 Hz, 1H), 3.69 – 3.56 (m, 20H), 3.53 (t, *J* = 5.4 Hz, 2H), 3.35 (t, *J* = 5.5 Hz, 2H), 3.20 (ddd, *J* = 8.9, 5.8, 4.4 Hz, 1H), 2.92 (dd, *J* = 12.7, 5.0 Hz, 1H), 2.70 (d, *J* = 12.7 Hz, 1H), 2.21 (t, *J* = 7.6 Hz, 2H), 1.81 – 1.53 (m, 4H), 1.51 – 1.38 (m, 2H). **^13^C NMR** (100 MHz, CD_3_OD) δ 176.1, 168.9, 166.1, 137.2, 133.1, 129.2, 127.7, 124.8, 71.6 (2C), 71.5, 71.3, 71.2, 70.6, 70.5, 63.4, 61.6, 57.0, 41.1, 41.0, 40.4, 36.7, 29.8, 29.5, 26.8. **ESI MS**: 741 ([M + Na]^+^). **HR ESI MS**: calcd for C_31_H_45_O_8_N_6_F_3_NaS 741.28639, found 741.28698.

**Riboflavin tetraacetate (**Compound **2)**

Compound **2** was prepared as described in the literature, and the spectroscopic data were in accordance with the reported data.^2^

**Riboflavin tetraacetate-C_6_-NHBoc (**Compound **3)**

Compound **3** was prepared analogously to a procedure described in the literature.^2^ Compound **2** (50 mg, 92 μmol, 1.0 eq.) and *N*-Boc-6-bromo-hexylamine (129 mg, 460 μmol, 5.0 eq.) were dissolved in anhydrous DMF (3 mL) under an inert atmosphere. Cesium carbonate (46 mg, 140 μmol, 1.5 eq.) was then added, and the reaction mixture was stirred in the dark for 4.5 hours. Next, the reaction was quenched by addition of H_2_O (10 mL) and saline (10 mL), and the product was extracted with DCM (3 × 15 mL). The combined organic extracts were dried over MgSO_4_ and the volatiles were evaporated. The product was purified by column chromatography (mobile phase DCM:MeOH / 20:1) to yield **3** as a bright orange oil (60 mg, 90%).

**^1^H NMR** (400 MHz, CDCl_3_) δ 8.01 (s, 1H), 7.52 (s, 1H), 5.70 – 5.61 (m, 1H), 5.49 – 5.43 (m, 1H), 5.42 – 5.36 (m, 1H), 5.14 – 4.71 (m, 1H), 4.56 – 4.48 (m, 1H), 4.42 (dd, *J* = 12.4, 2.9 Hz, 1H), 4.23 (dd, *J* = 12.3, 5.8 Hz, 1H), 4.08 – 4.00 (m, 2H), 3.14 – 3.02 (m, 2H), 2.54 (s, 3H), 2.42 (s, 3H), 2.29 (s, 3H), 2.21 (s, 3H), 2.06 (s, 3H), 1.78 – 1.63 (m, 7H), 1.53 – 1.30 (m, 13H). **^13^C NMR** (100 MHz, CDCl_3_) δ 170.6, 170.3, 169.9, 169.7, 159.6, 156.0, 155.0, 149.1, 147.4, 136.5, 135.8, 134.6, 132.9, 131.2, 115.3, 78.9, 70.4, 69.4, 69.0, 61.9, 44.4, 41.9, 40.5, 29.9, 28.4, 27.6, 26.6, 26.5, 21.4, 21.1, 20.8, 20.7, 20.3, 19.4. **ESI MS**: 766 ([M + Na]^+^). **HR ESI MS**: calcd for C_36_H_49_O_12_N_5_Na 766.32699, found 766.32667.

**Riboflavin tetraacetate-C_6_-NH_2_ (RFT)**

 Compound **3** (35 mg, 47 μmol, 1.0 eq.) was dissolved in DCM (1 mL) and TFA (1 mL). The reaction mixture was stirred for 15 min and the volatiles were removed. The product was purified by reversed-phase HPLC (C18, gradient 20% → 60% MeCN in H_2_O + 0.1% TFA) to yield **RFT** (24 mg, 69%) as an orange lyophilizate.

**^1^H NMR** (400 MHz, CD_3_OD) δ 7.96 (s, 1H), 7.86 (s, 1H), 5.72 – 5.64 (m, 1H), 5.54 (dd, *J* = 5.9, 4.5 Hz, 1H), 5.43 (td, *J* = 6.1, 3.1 Hz, 1H), 5.18 – 5.05 (m, 2H), 4.51 (dd, *J* = 12.3, 3.2 Hz, 1H), 4.25 (dd, *J* = 12.3, 6.2 Hz, 1H), 4.10 – 4.02 (m, 2H), 2.94 (t, *J* = 7.6 Hz, 2H), 2.62 (s, 3H), 2.48 (s, 3H), 2.20 (s, 3H), 2.19 (s, 3H), 2.03 (s, 3H), 1.80 – 1.62 (m, 7H), 1.55 – 1.41 (m, 4H). **^13^C NMR** (100 MHz, CD_3_OD) δ 170.9, 170.4, 170.1, 170.0, 160.4, 156.0, 149.4, 148.1, 137.2, 135.5, 134.8, 131.6, 131.3, 116.2, 70.3, 69.4, 69.3, 61.6, 44.4, 41.2, 39.2, 27.0, 25.9, 25.5, 19.9, 19.6, 19.3, 19.2, 18.9, 17.9. **ESI MS**: 644 ([M + H]^+^). **HR ESI MS**: calcd for C_31_H_42_O_10_N_5_ 644.29262, found 644.29241.

**2-(4'-(2-methoxypropan-2-yl)-[2,2'-bipyridin]-4-yl)propan-2-ol (**Compound **4)**

Compound **4** was prepared according as described in the literature, and the spectroscopic data were in accordance with the reported data.^3^

**4-(20-azido-2-methyl-3,6,9,12,15,18-hexaoxaicosan-2-yl)-4'-(2-methoxypropan-2-yl)-2,2'-bipyridine (**Compound **5)**

Compound **4** (25 mg, 87 μmol, 1.0 eq.) was dissolved in anhydrous DMF (2 mL) under an inert atmosphere, and NaH (3.1 mg, 131 μmol, 1.5 eq.) was added. The reaction mixture was stirred for 30 minutes before N_3_-PEG-5-OTs (40 mg, 87 μmol, 1 eq.) was added. The reaction mixture was then heated to 55 °C for 48 hours in a sealed ampoule. The product was then purified by reversed-phase HPLC (C18, gradient 30% → 80% MeCN in H_2_O) to yield **5** (19 mg, 38%) as a colorless oil.

**^1^H NMR** (400 MHz, CDCl_3_) δ 8.64 (dd, *J* = 5.2, 2.4 Hz, 2H), 8.38 (d, *J* = 1.8 Hz, 2H), 7.45 (dd, *J* = 5.1, 1.8 Hz, 1H), 7.38 (dd, *J* = 5.2, 1.8 Hz, 1H), 3.68 – 3.60 (m, 20H), 3.40 – 3.33 (m, 4H), 3.13 (s, 3H), 1.58 (s, 6H), 1.57 (s, 6H). **^13^C NMR** (101 MHz, CDCl_3_) δ 156.7, 156.6, 156.5, 156.4, 149.4, 149.3, 121.1, 121.0, 118.4, 118.4, 76.5, 70.8, 70.7, 70.7, 70.6, 70.6, 70.0, 62.6, 51.0, 50.7, 27.9, 27.4. **ESI MS**: 576 ([M + H]^+^). **HR ESI MS**: calcd for C_29_H_44_O_7_N_5_ 576.33918, found 576.33924.

**Compound 6**

Compound **6** was prepared as described in the literature, and the spectroscopic data were in accordance with the reported data.^3^

**N_3_-PEG_5_-Ir-catalyst (Ir cat)**

Compounds **6** (20 mg, 23 μmol, 1.0 eq.) and **5** (13 mg, 23 μmol, 1.0 eq.) were dissolved in a 1:1 mixture of DCM:MeOH (4 mL), and the reaction mixture was stirred overnight in the dark at room temperature. The volatiles were then removed, and the residue was purified by reversed-phase HPLC (C18, gradient 30% → 80% MeCN in H_2_O + 0.1% TFA) to yield **Ir** **cat** (15 mg, 48%) as a yellow greenish oil.

**^1^H NMR** (400 MHz, CD_3_OD) δ 8.87 (d, *J* = 1.8 Hz, 1H), 8.82 (d, *J* = 1.8 Hz, 1H), 8.37 (d, *J* = 2.8 Hz, 2H), 8.08 (dd, *J* = 5.8, 2.4 Hz, 2H), 7.92 (dd, *J* = 5.8, 1.8 Hz, 1H), 7.80 (dd, *J* = 5.8, 1.8 Hz, 1H), 7.49 (d, *J* = 2.0 Hz, 2H), 6.85 – 6.71 (m, 2H), 5.86 (dt, *J* = 8.2, 2.5 Hz, 2H), 3.72 – 3.52 (m, 20H), 3.48 (t, *J* = 4.5 Hz, 2H), 3.35 (t, *J* = 5.0 Hz, 2H), 3.23 (s, 3H), 1.67 (s, 3H), 1.66 (s, 3H), 1.64 (s, 3H), 1.64 (s, 3H). **^19^F NMR** (377 MHz, CD_3_OD) δ -61.87 (d, *J* = 25.9 Hz, 6F), -76.9 (s, 3F), -104.3 (m, 2F), -108.1 (m, 2F). **ESI MS**: 1372 ([M]^+^). **HR ESI MS**: calcd for C_55_H_55_O_11_N_7_F_10_Ir 1372.34236, found 1372.34193.

**Synthesis of monomers, polymer precursors, and polymer conjugates (iBodies)**

**Materials**

# 1-Amino-propan-2-ol, methacrylic anhydride, *β-*alanine, 4,5 dihydrothiazole-2-thiol (TT), *N*-ethyl-*N*′-(3-dimethylaminopropyl)carbodiimide hydrochloride (EDC), carbon disulfide, ethanethiol, sodium hydride (60% dispersion in mineral oil), *N*,*N*-diisopropylethylamine (DIPEA), *tert*-butanol, *N*,*N*-dimethyl acetamide (DMAA), dimethyl sulfoxide (DMSO), and *N*-biotinyl-ethylenediamine trifluoroacetate salt (NH_2_-biotin) were purchased from Merck. HABA/Avidin Reagent kit was purchased from Merck (Czech Republic). The initiators 2,2′-azobis(2-methylpropionitrile) (AIBN) and 2,2′-azobis(4-methoxy-2,4-dimethylvaleronitrile) (V-70) were purchased from Wako Chemicals (Japan). ATTO488-amine was purchased from ATTO-TEC (Germany). NH_2_-DBCO and DBCO-COOH were obtained from Click Chemistry Tools (USA).

# Sephadex LH-20 and PD10 columns were purchased from Cytiva (Sweden). All other chemicals and solvents were of analytical grade.

**Synthesis of monomers and chain transfer agents**

*N*-(2-Hydroxypropyl)methacrylamide (HPMA) monomers were synthesized as previously described.^4^ 3-(3-Methacrylamidopropanoyl)thiazolidine-2-thione (Ma-*β-*Ala-TT) monomer was prepared by a two-step procedure. First, 3-methacrylamidopropanoic acid (Ma-*β-*Ala-COOH) was synthesized by reacting *β-*alanine with methacrylic anhydride. Briefly, *β-*alanine (10 g, 0.112 mol) was suspended in 100 mL acetonitrile with 10 mL distilled water. A small amount of inhibitor (octylpyrocatechin) was added to the solution. The mixture was heated to 40 ℃, and methacrylic-anhydride (11.3 g, 0.117 mol) in 15 mL of acetonitrile was added dropwise. After addition of methacrylic-anhydride, a clear solution formed. The reaction mixture was stirred for 6 hours at 40 ℃. Acetonitrile and water were evaporated *in vacuo*. The residue was diluted with 150 mL diethyl ether and allowed to crystallize in a freezer. Crystallized Ma-*β-*Ala-COOH was filtered off, washed twice with 50 mL diethyl ether, and dried. In the second step, Ma-*β-*Ala-TT was prepared by reacting Ma-*β-*Ala-COOH with 4,5-dihydrothiazole-2-thiol (TT) in the presence of EDC as previously described.^5^

The chain transfer agents S-2-cyano-2-propyl S′-ethyl trithiocarbonate (CTA-AIBN) and *N*-(3-azidopropyl)-4-cyano-4-ethylsulfanylcarbothioylsulfanyl-pentanamide (CTA-N_3_) were synthesized as previously described.^6,7^

**Preparation of azide-modified HRP**

Horseradish peroxidase (HRP, Type VI-A, Merck P6782) was dissolved in buffer M (200 mM pyridoxal phosphate, 50 mM sodium phosphate, pH 6.5) to a final concentration of 1 mg/mL and incubated overnight at 4 °C.^8^ The aldehyde-modified protein was then purified by six rounds of ultrafiltration using Amicon 10 kDa MWCO units, transferring it into buffer T [20 mM 2-amino-5-methoxybenzoic acid (AMB), 50 mM sodium citrate, pH 4.5] while maintaining the same concentration. The purified protein was reacted overnight at 4 °C with a 10-fold molar excess of N3-PEG5-hydroxyamine.^9^ AMB was pre-purified by RP-HPLC to remove violet polymer contaminants formed during storage. The resulting azide-modified protein was purified by six additional rounds of ultrafiltration using Amicon 10 kDa MWCO units, transferring it into buffer S (50 mM sodium phosphate, pH 6.5) to a final concentration of 2 mg/mL. Aliquots of the enzyme were shock-frozen in liquid nitrogen and stored at -80 °C until use.

**Synthesis of polymer precursors**

pHPMA-co-Ma-*β-*Ala-TT copolymer precursors (P1–P6) used for the synthesis of iBodies **1-6** were prepared by reversible addition-fragmentation chain-transfer copolymerization (RAFT) of monomers HPMA and Ma-*β-*Ala-TT in the presence of the chain transfer agent CTA-AIBN or CTA-N_3_ and the initiator 2,2′-azobis(2-methylpropionitrile) (AIBN) or 2,2’-azobis(4-methoxy2,4-dimethylvaleronitrile (V-70). Detailed procedures are listed in the corresponding iBody synthesis description.

**Synthesis of iBodies**

**Synthesis of** HRP iBody 1

To prepare the P1 precursor, 1.0 g (6.98 × 10^-3^ mmol) of HPMA (90 %mol) and 0.200 g (0.776 × 10^-3^ mmol) of Ma-*ß*-Ala-TT (10 %mol) were dissolved in 0.85 mL DMSO. A 4.47 mg aliquot (1.29 × 10^-2^ mmol) of the chain transfer agent CTA-N_3_, 1.99 mg (6.47 × 10^-3^ mmol) of V-70, and 8.2 mL of *tert*-butanol were added. The ratio of monomers to chain transfer agent to initiator (M:CTA:I) was 1200:2:1. The solution was introduced into a polymerization ampoule. Before sealing, the mixture was bubbled with argon for 10 min. Polymerization was carried out at 40 °C for 16 hours. The polymer precursor was isolated by precipitation into a 3:1 mixture of acetone:diethyl ether, filtered off, washed with acetone and diethyl ether, and dried in a vacuum. The terminating trithiocarbonate group was removed according to the procedure described by Perrier *et al*.^10^ The final polymer precursor P1 was isolated by the precipitation procedure described above. The yield of P1 was 607 mg, and it had the following characteristics: *M_w_* = 67 000 g mol^-1^, *Ð* = 1.12, content of TT reactive groups was 10.3 %mol.

**HRP iBody** **1** was synthesized by a two-step procedure. In the first step, the copolymer precursor P1 (8.0 mg, 5.14 × 10^−2^ mmol TT groups) was dissolved in DMSO (300 μL) and reacted with GCPII inhibitor (xGCPII, Šácha *et al.^11^*, compound 1) (1.1 mg, 1.23 × 10^−3^ mmol), DBCO-COOH (0.04 mg, 1.20 × 10^−4^ mmol), and *N*,*N*-diisopropylethylamine (DIPEA) (1.5 μL, 8.69 ×10^−3^ mmol). The reaction was carried out for 4 h at room temperature. The TT reactive groups were incubated with DBCO-NH_2_ (0.6 mg, 2.17 ×10^−3^ mmol) for 4 h at room temperature, resulting in partial reaction. Residual TT reactive groups were removed by reaction with 1-amino-propan-2-ol (5 µL, 6.48 × 10^−2^ mmol), and the reaction was stirred for the next 10 min. The reaction mixture was diluted with 1 mL of methanol and purified on a 1.5×18 cm chromatography column filled with Sephadex LH-20 in methanol. Product-containing fraction was detected at 220 nm with an Azura UVD 2.1S UV-Vis detector (Knauer). Methanol was evaporated. The modified P1 precursor was dissolved in Milli-Q water, purified on a PD10 column, and lyophilized. The yield of modified precursor P1 was 9 mg, *M*_w_’ = 77 400 g mol^-1^. The content of xGCPII was 8.45 wt%.

In the second step, the modified P1 precursor containing DBCO reactive groups (14.0 per polymer) and xGCPII (8.4 per polymer) was incubated overnight at 4 °C with an equimolar amount of azide-modified HRP relative to the DBCO content in PBS buffer. The polymer conjugate was then purified by six rounds of ultrafiltration using Amicon 100 kDa MWCO units in the same buffer. The degree of HRP substitution per polymer (5.5 HRP molecules per polymer) was determined by amino acid analysis. The conjugate was stored at 4 °C until use. The calculated molar mass of the final conjugate is *M*_w_’ = 319 000 g mol^-1^.

**Synthesis of** Ir cat iBody 2

To prepare the P2 precursor, 1.2 g (8.38 × 10^-3^ mmol) of HPMA (88 %mol) and 0.295 g (1.143 × 10^-3^ mmol) of Ma-ß-Ala-TT (12 %mol) were dissolved in 1.78 mL DMSO. A 3.01 mg aliquot (1.47 × 10^-2^ mmol) of the chain transfer agent CTA-AIBN, 2.26 mg (7.33 × 10^-3^ mmol) of 2,2′-azobis(4-methoxy-2,4-dimethylvaleronitrile) (V-70), and 11.6 mL of *tert*-butanol were added. The M:CTA:I ratio was 1300:2:1. The solution was introduced into a polymerization ampoule. Before sealing, the mixture was bubbled with argon for 10 min. Polymerization was carried out at 40 °C for 16 h.

The polymer precursor P2 was isolated using the same procedure as P1. The yield of P2 was 870 mg. It had the following characteristics: *M*_w_ = 66 000 g mol^-1^, *Ð* = 1.16, and the content of TT reactive groups was 13.3 %mol.

**Ir cat iBody** **2** was synthesized by a two-step procedure. In the first step, the P2 copolymer precursor (9.07 mg, 7.61 × 10^−3^ mmol TT groups) was reacted with xGCPII (2.0 mg, 2.34 × 10^−3^ mmol) and NH_2_-DBCO (0.93 mg, 3.03 × 10^−3^ mmol) dissolved in DMSO (300 μL). Then, *N*,*N*-diisopropylethylamine (DIPEA) (4.6 μL, 5.27 ×10^−3^ mmol) was added. The reaction was carried out for 4 h at room temperature. Residual TT reactive groups were removed by reaction with 1-amino-propan-2-ol (2 µL, 2.59 × 10^−2^ mmol), and the reaction was stirred for the next 10 min. The modified P2 precursor with attached xGCPII and DBCO was diluted with 1 mL of methanol and purified on a 1.5×18 cm chromatography column filled with Sephadex LH-20 in methanol. Product-containing fraction was detected at 220 nm with an Azura UVD 2.1S UV-Vis detector (Knauer). Methanol was evaporated. The modified P2 precursor was dissolved in Milli-Q water, purified on a PD10 column, and lyophilized.

In the second step, P2 with attached xGCPII and DBCO (6.25 mg) was reacted with **Ir cat** (2.69 mg, 3.03 × 10^−3^ mmol) in DMSO (100 μL) for 6 h at room temperature. The reaction mixture with **Ir cat iBody** **2** was diluted with 1 mL of methanol, and **Ir cat iBody** **2** was purified by column chromatography as described above.

The yield of **Ir cat iBody** **2** was 7 mg. The content of xGCPII was 10.73 wt%; Ir cat was 10.99 wt%, *M*_w_’ = 79 200 g mol^-1^.

**Synthesis of** RFT iBody 3

The P3 precursor was prepared under the same conditions as P2.

The yield of P3 was 850 mg; *M*_w_ = 62 000 g mol^-1^, *Ð* = 1.05, and content of TT reactive groups = 13.3 %mol.

P3 (8 mg, 6.71 × 10^−3^ mmol TT groups), xGCPII (1.4 mg, 1.57 × 10^−3^ mmol), and **RFT** (1.7 mg, 2.24 × 10^−3^ mmol) were dissolved in DMSO (300 μL), and then *N*,*N*-diisopropylethylamine (DIPEA) (3.3 μL, 3.81 × 10^−3^ mmol) was added. The reaction was carried out for 4 h at room temperature. Residual TT reactive groups were removed by reaction with 1-amino-propan-2-ol (2 µL, 2.59 × 10^−2^ mmol), and the reaction was stirred for the next 10 min. The reaction mixture was diluted with 1 mL of methanol, and **RFT iBody 3** was isolated by the same procedure as **Ir cat iBody** **2**. The yield of **RFT iBody 3** was 10 mg. The content of xGCPII was 10.25 wt%; RFT was 11.8 wt%, *M*_w_’ = 79 500 g mol^-1^.

**Synthesis of** iBody 4

To prepare P4, 0.5 g (3.49 × 10^-3^ mmol) of HPMA (92 %mol) and 0.078 g (0.304 × 10^-3^ mmol) of Ma-ß-Ala-TT (8 %mol) were dissolved in 0.84 mL DMSO. A 1.29 mg aliquot (5.84 × 10^-3^ mmol) of the chain transfer agent S-2-cyano-2-propyl S′-ethyl trithiocarbonate, 0.48 mg (2.94 × 10^-3^ mmol) of the initiator 2,2′-azobis(2-methylpropionitrile) (AIBN), and 3.1 mL of *tert*-butanol were added. The M:CTA:I ratio was 1300:2:1. The solution was introduced into a polymerization ampoule. Before sealing, the mixture was bubbled with argon for 10 min. Polymerization was carried out at 70 °C for 16 h. The P4 polymer precursor was isolated by the procedure described above. The yield of P4 was 380 mg; *M*_w_ = 72 000 g mol^-1^, *Ð* = 1.20, and content of TT reactive groups = 5.4 %mol.

P4 (50 mg, 18.07 × 10^−3^ mmol TT groups), xGCPII (3.12 mg, 4.1 × 10^−3^ mmol), and ATTO488 (3.33 mg, 3.88 × 10^−3^ mmol) were dissolved in DMSO (400 μL), and then *N*,*N*-diisopropylethylamine (DIPEA) (4.9 μL, 2.81 ×10^−2^ mmol) was added. The reaction was carried out for 4 h at room temperature. Residual TT reactive groups were removed by reaction with 1-amino-propan-2-ol (5 µL, 6.48 × 10^−2^ mmol), and the reaction was stirred for the next 10 min. The reaction mixture was diluted with 1 mL of methanol, and **iBody** **4** was isolated by the same procedure as **Ir cat** **iBody** **2**.

The yield of **iBody** **4** was 40 mg. The content of xGCPII was 13.5 wt%; of ATTO488 was 4.39 wt%, *M*_w_’ = 87 700 g mol^-1^.

**Synthesis of** iBody 5

To synthesize P5, 0.5 g (3.49 × 10^-3^ mmol) of HPMA (90 %mol) and 0.100 g (0.388 × 10^-3^ mmol) of Ma-ß-Ala-TT (10 %mol) were dissolved in 0.84 mL DMSO. A 4.29 mg aliquot (1.94 × 10^-2^ mmol) of the chain transfer agent CTA-AIBN, 1.59 mg (9.70 × 10^-3^ mmol) of the initiator AIBN, and 4.3 mL of *tert*-butanol were added. The M:CTA:I ratio was 400:2:1. The solution was introduced into a polymerization ampoule. Before sealing, the mixture was bubbled with argon for 10 min. Polymerization was carried out at 70 °C for 16 h. P5 was isolated by the procedure described above. The yield of P5 was 410 mg; *M*_w_ = 26 600 g mol^-1^, *Ð* = 1.07, and content of TT reactive groups = 10.4 %mol.

P5 (70 mg, 4.52 × 10^−2^ mmol TT groups), xGCPII (10.0 mg, 1.29 × 10^−2^ mmol), and NH_2_-biotin (5.0 mg, 1.75 × 10^−2^ mmol) were dissolved in DMSO (400 μL), and then *N*,*N*-diisopropylethylamine (DIPEA) (11.2 μL, 6.43 ×10^−2^ mmol) was added. The reaction was carried out for 4 h at room temperature. Residual TT reactive groups were removed by reaction with 1-amino-propan-2-ol (5 µL, 6.48 × 10^−2^ mmol), and the reaction was stirred for the next 10 min. The reaction mixture was diluted with 1 mL of methanol, and **iBody** **5** was isolated by the same procedure as **Ir cat iBody** **2**. The yield of **iBody** **5** was 60 mg. The content of xGCPII was 11.5 wt%; of NH_2_-biotin was 2.5 wt%, *M*_w_’ = 30 900 g mol^-1^.

**Synthesis of** iBody 6

To synthesize P6, 1.0 g (6.98 × 10^-3^ mmol) of HPMA (88 %mol) and 0.246 g (0.952 × 10^-3^ mmol) of Ma-ß-Ala-TT (12 %mol) were dissolved in 1.584 mL DMSO. A 2.34 mg aliquot (1.06 × 10^-2^ mmol) of the chain transfer agent CTA-AIBN, 0.869 mg (5.29 × 10^-3^ mmol) of AIBN, and 7.1 mL of *tert*-butanol were added. The M:CTA:I ratio was 1500:2:1. The solution was introduced into a polymerization ampoule. Before sealing, the mixture was bubbled with argon for 10 min. Polymerization was carried out at 70 °C for 16 h. P6 was isolated by the procedure described above. The yield of P6 was 630 mg; *M*_w_ = 78 500 g mol^-1^, *Ð* = 1.19, and content of TT reactive groups = 12.9 %mol.

P6 (30 mg, 2.45 × 10^−2^ mmol TT groups) and NH_2_-biotin (2.67 mg, 7.26 × 10^−3^ mmol) were dissolved in DMSO (300 μL), and then *N*,*N*-diisopropylethylamine (DIPEA) (1.9 μL, 1.09 ×10^−2^ mmol) was added. The reaction was carried out for 4 h at room temperature. Residual TT reactive groups were removed by reaction with 1-amino-propan-2-ol (5 µL, 6.48 × 10^−2^ mmol), and the reaction was stirred for the next 10 min. The reaction mixture was diluted with 1 mL of methanol, and **iBody** **6** was isolated by the same procedure as **Ir cat iBody** **2**. The yield of **iBody** **6** was 20 mg. The content of NH_2_-biotin was 3.45 wt%, *M*_w_’ = 81 300 g mol^-1^.

**Characterization of polymer precursors and iBodies**

The weight-average molecular weights (*M*_w_), number-average molecular weights (*M*_n_), and dispersities (*Đ*) of the polymer precursors P1-P6 were determined using a Shimadzu HPLC system equipped with a UV detector, an Optilab rEX differential refractometer, a DAWN 8 multiangle light scattering detector (Wyatt Technology, USA), and a TSKgel G4000SWXL size-exclusion chromatography column. The *M*_w_, *M*_n_, and *Đ* were calculated using Astra V software. The refractive index increment dn/dc = 0.167 mL/g was used for calculation. The mobile phase contained 300 mM sodium acetate buffer, pH 6.5, and methanol (20%/80% v/v). The flow rate was 0.5 ml/min. The content of TT reactive groups in the polymer precursors was determined spectrophotometrically (ε_305nm_ = 10 600 L mol^−1^ cm^−1^, methanol). The content of xGCPII (1 Br atom per a compound molecule) in iBodies **1-5** and the content of Ir cat (1 Ir atom per a compound molecule) in **Ir cat iBody** **2** was determined by inductively coupled plasma atomic emission spectroscopy (ICP-AES) using a Spectro ARCOS MultiView (SPECTRO Analytical Instruments, Kleve, BRD). The content of RFT in **RFT** **iBody** **3** (ε_371nm_ = 8,800 L mol^−1^ cm^−1^, water) and the content of the fluorophore ATTO488 in **iBody** **4** (ε_502nm_ = 90 000 L mol^−1^ cm^−1^, water) were determined using a Specord 205 spectrophotometer (Analytik Jena, Germany). The content of NH_2_-biotin in **iBodies** **5** and **6** was determined using the HABA/Avidin Reagent kit (Merck) for spectrophotometric determination at 500 nm according to the manufacturer’s instructions (Sigma, H 2135). *M*_w_' for iBodies was calculated as an increase in precursor *M*_w_ by weight percent of bound molecules.

**Confocal microscopy**

GCPII-transfected human glioblastoma cell line U251 MG, which allows switchable expression of GCPII (Tet-OffAdvanced System), was prepared as previously described by Neburkova *et al.*^12^ U251 MG or U251 MG-GCPII cells were grown in DMEM – high glucose (Sigma-Aldrich D6429) and 10% FBS (Gibco 10270-106) to 30% confluence in 35-mm 4-chamber dishes with glass bottoms (Bio-Port Europe s.r.o.). After two days, fresh high glucose, no phosphate DMEM (ThermoFisher Scientific 11971025) with internalization inhibitors MDC (monodansyl cadaverine), LY 294002, or PitStop 2 in final concentrations of 200 µM, 50 µM, or 20 µM, respectively, was added. Cells were incubated at 37 °C for 30, 60, and 20 min, respectively. **iBody 4** solution was added to the media to a final concentration of 100 nM, and the cells were incubated for 1 h at 37 °C. The solutions were then replaced with fresh TBS.

Confocal images (pinhole 1 Airy unit) of cells in each chamber were taken at room temperature with a Zeiss LSM 780 confocal microscope (Carl Zeiss Microscopy) equipped with a spectral detector and an oil-immersion objective (Plan-Apochromat 63x/1.40 Oil DIC M27). The excitation was performed using a 488 nm argon-ion laser (max. power 25 mW) with emission collected from 499 to 578 nm (1% power, voltage on detector: 800 V). Images were acquired with ZEN 2011 software and processed with the Fiji image processing package.

**Proximity-selective** **labeling on live U-251 MG–GCPII** **cells for Western blot and quantitative LC-MS/MS analyses**

**Proximity-selective** HRP iBody 1 **labeling and untargeted free HRP** **labeling**

Each sample contained 40 million U-251 MG^+^ cells expressing GCPII.^12^ Cells were detached from the dish and resuspended in DMEM – high glucose (Sigma-Aldrich D6429) and 10% FBS (Gibco 10270-106). Cell suspensions were transferred to 2 mL Eppendorf tubes and centrifuged at 250 rcf for 3 min at RT, then washed twice in TBS and pelleted. Pellets were resuspended in 995 µL or 975 µL of 20 µM PitStop 2 for the samples and control, respectively, to prevent internalization of GCPII bound to iBody. The cells were incubated on a rotisserie for 20 min at RT. Then, 20 µL of 100 mM 2-(phosphonomethyl)pentanedioic acid 2-PMPA, a specific GCPII inhibitor, was added to the control samples to a final concentration of 2 mM, and they were left to incubate for 15 min. For targeted biotinylation, 5 µL of 10 µM **HRP iBody 1** in TBS was added to a final concentration of 50 nM, and samples were incubated on a rotisserie for 20 min at RT. The cells were centrifuged to remove the supernatant, typically at 300 rcf for 3 min at RT, washed twice with 1 mL 20 µM PitStop in TBS, and resuspended in 1 mL of reaction buffer (0.2 mM biotinyl-tyramide, 20 µM PitStop, and 0.03% H_2_O_2_ in TBS) as previously described.^13^ Free nonconjugated HRP (HRP, Type VI-A, Merck P6782) at a concentration of 0.1 mg/mL in the presence of 0.2 mM biotinyl-tyramide, 20 µM PitStop, and 0.03% H_2_O_2_ in TBS was used for untargeted labeling. A control reaction was performed in the absence of H_2_O_2_. After 5 min incubation on a rotisserie, cells were pelleted and washed twice with 1 mL TBS. The cell pellets were then lysed in 200 μL of lysation solution (1% Tween 20 in TBS, 1 tablet Complete protease inhibitors per 10 mL). The samples were sonicated for 5 min on ice using an Elmasonic S 30. Then, the samples were left 10 min on ice and spun at 15,000 rcf for 10 min at 4 °C. Pellets were discarded. The supernatant protein concentration was measured by Bradford protein assay. The samples were then diluted to 1.3 mL with TBS and added to a 1.5 mL Eppendorf tube containing 60 μL of Streptavidin Agarose Ultra Performance (VectorLabs, N-1000) pre-washed twice with 1 mL TBS buffer. The samples were incubated overnight at 4 °C on a rotisserie, and the beads were pelleted at 1,000 rcf for 1 min at 4 °C. The supernatant was removed, and the beads were washed 6x with 1 mL TBS. After the final wash, proteins were eluted with reducing SDS sample buffer and heated to 95 °C for 5 min. Protein eluates obtained from six replicates were analyzed using LC–MS/MS, and pooled replicate eluates were employed for Western blot analysis.

**Proximity-selective** Ir cat iBody 2 **labeling and untargeted free** Ir cat **labeling**

Each sample contained 40 million U251 MG–GCPII. Cells were detached from the dish and resuspended in DMEM – high glucose (Sigma-Aldrich D6429) and 10% FBS (Gibco 10270-106). Cell suspensions were transferred to 2 mL Eppendorf tubes and centrifuged at 250 rcf for 3 min at RT, then washed twice in TBS and pelleted. Pellets were resuspended in 995 µL or 975 µL of 20 µM PitStop 2 for the samples and control, respectively. The cells were incubated on a rotisserie for 20 min at RT. Then, 20 µL of 100 mM 2-PMPAwas added to the control samples to a final concentration of 2 mM and they were left to incubate 15 min. For targeted biotinylation, 5 µL of 10 µM **Ir cat iBody 2** was added to a final concentration of 50 nM and incubated on a rotisserie for 20 min at RT. The cells were centrifuged to remove the supernatant, typically at 300 rcf for 3 min at RT, washed twice with 1 mL 20 µM PitStop in TBS, and resuspended in 1 mL of TBS containing 0.25 mM diazirine biotin PEG 5 (**1**) and 20 µM PitStop 2. Free nonconjugated 250 µM **Ir cat** in the presence of 0.25 mM **1** and 20 µM PitStop 2 was used for untargeted labeling. The samples were transferred to Liquid scintillation vials (Sigma-Aldrich, Z190527), placed in irradiation cells, and irradiated with 450-nm blue light for 10 min as previously described.^3^ A reaction in the absence of irradiation was performed as a control for untargeted labeling. Then, the cells were pelleted and washed twice with 1 mL TBS. The cell pellets were then lysed in 200 μL of lysation solution (1% Tween 20 in TBS, 1 tablet Complete protease inhibitors per 10 mL). The samples were then sonicated for 5 min on ice using an Elmasonic S 30. The samples were left 10 min on ice and spun at 15,000 rcf for 10 min at 4 °C. Pellets were discarded. The supernatant protein concentration was measured by Bradford protein assay. The samples were then diluted to 1.3 mL with TBS and added to a 1.5 mL Eppendorf tube containing 60 μL of Streptavidin Agarose Ultra Performance (VectorLabs, N-1000) pre-washed twice with 1 mL TBS buffer. The samples were incubated overnight at 4 °C on a rotisserie, and the beads were then pelleted at 1,000 rcf for 1 min at 4 °C. The supernatant was removed, and the beads were washed 6x with 1 mL TBS. After the final wash, proteins were eluted with reducing SDS sample buffer and heated to 95 °C for 5 min. Protein eluates obtained from six replicates were analyzed using LC–MS/MS, and pooled replicate eluates were employed for Western blot analysis.

**Proximity-selective** RFT iBody 3 **labeling and untargeted free RFT** **labeling**

Each sample contained 40 million U251 MG–GCPII. Cells were detached from the dish and resuspended in DMEM – high glucose (Sigma-Aldrich D6429) and 10% FBS (Gibco 10270-106). Cell suspensions were transferred to 2 mL Eppendorf tubes and centrifuged at 250 rcf for 3 min at RT, then washed twice in TBS and pelleted. Pellets were resuspended in 995 µL or 975 µL of 20 µM PitStop 2 for the samples and control, respectively. The cells were incubated on a rotisserie for 20 min at RT. Then, 20 µL of 100 mM 2-PMPA was added to the control samples to a final concentration of 2 mM, and they were left to incubate 15 min. For targeted biotinylation, 5 µL of 10 µM **RFT iBody 3** was added to a final concentration of 50 nM and incubated on a rotisserie for 20 min at RT. The cells were centrifuged to remove the supernatant, typically at 300 rcf for 3 min at RT, washed twice with 1 mL 20 µM PitStop in TBS, and resuspended in 1 mL of TBS containing 0.2 mM biotinyl-tyramide and 20 µM PitStop 2. Free nonconjugated 250 µM **RFT** in the presence of 0.2 mM biotinyl-tyramide and 20 µM PitStop 2 was used for nontargeted labeling. The samples were transferred to Liquid scintillation vials (Sigma-Aldrich, Z190527), placed in irradiation cells, and irradiated with 400-nm violet light for 5 min as previously described.^14^ A reaction in the absence of irradiation was performed as a control for untargeted labeling. Then, the cells were pelleted and washed twice with 1 mL TBS. The cell pellets were lysed in 200 μL of lysation solution (1 % Tween 20 in TBS, 1 table Complete protease inhibitors per 10 mL). The samples were then sonicated for 5 min on ice using an Elmasonic S 30. Then, the samples were left 10 min on ice and spun at 15,000 rcf for 10 min at 4 °C. Pellets were discarded. The supernatant protein concentration was measured by Bradford protein assay. The samples were then diluted to 1.3 mL with TBS and added to a 1.5 mL Eppendorf tube containing 60 μL of Streptavidin Agarose Ultra Performance (VectorLabs, N-1000) pre-washed twice with 1 mL TBS. The samples were incubated over night at 4 °C on a rotisserie, and the beads were pelleted at 1,000 rcf for 1 min at 4 °C. The supernatant was removed, and the beads were washed 6x with 1 mL TBS. After the final wash, proteins were eluted with reducing SDS sample buffer and heated to 95 °C for 5 min. Protein eluates obtained from six replicates were analyzed using LC–MS/MS, and pooled replicate eluates were employed for Western blot analysis.

**Liquid chromatography-tandem mass spectrometry (LC-MS/MS) data acquisition**

Proteins were treated with TCEP and chloroacetamide (65 °C, 30 min) to reduce and alkylate cysteines. Samples were placed on filters (Microcon-10 kDa Centrifugal Filter)^15^ and digested with 0.1 μg of trypsin solution in 50 mM ammonium bicarbonate at 37 °C for 16 h.

LC separation was performed using a Dionex Ultimate 3000 nano-HPLC system coupled to an Orbitrap Fusion Lumos mass spectrometer (Thermo Scientific). Samples were loaded onto a trap column (C18 PepMap100, 5 μm particle size, 300 μm × 5 mm; Thermo Scientific). The loading buffer consisted of water, 2% acetonitrile, and 0.1% trifluoroacetic acid. Mobile phase A consisted of water and 0.1% formic acid, and mobile phase B consisted of acetonitrile and 0.1% formic acid. Peptides were eluted with a gradient from 5% to 35% B over 53 min (in data-dependent acquisition, DDA, mode) or 100 min (in data-independent acquisition, DIA, mode). For LC/MS analysis, a nano reversed-phase column (EASY-Spray, 50 cm × 75 μm ID, PepMap C18, 2 μm particles, 100 Å pore size; Thermo Scientific) was used.

In DDA mode, MS1 scans of peptide precursors were acquired in the Orbitrap over the 350–1400 m/z range at 120 K resolution with the following settings: RF Lens 60%, maximum injection time 246 ms, and AGC target 250%. Only precursors with charge states 2–7 and intensity above 5000 were selected for fragmentation. The dynamic exclusion duration was set to 20 s with a 10 ppm tolerance. Peptide precursors were isolated by the quadrupole using a 1.2 m/z isolation window and fragmented by HCD with 28% normalized collision energy. Fragment ions were detected in the ion trap with the normalized AGC target set to 1000% and a maximum injection time of 35 ms.

In DIA mode, MS1 scans were acquired in the Orbitrap over the 350–1300 m/z range at 60 K resolution with the following settings: RF Lens 30%, maximum injection time 100 ms, and AGC target 100%. DIA scans were performed in the Orbitrap at 30 K resolution, with AGC target set to 1000% and maximum injection time mode set to Custom. HCD fragmentation spectra were acquired for ions within the m/z range of 400–1000 using variable isolation windows: Δm/z = 16 for 400–500, Δm/z = 8 for 500–650, and Δm/z = 16 for 650–1000.

The mass spectrometry proteomics data have been deposited to the ProteomeXchange Consortium^16^ via the PRIDE^17^ partner repository with the dataset identifier PXD068378.

**Protein identification and quantification**

DIA-NN (version 1.8.1) was used for library-free DIA analysis. ^18^ All settings were set as default, and match between runs was enabled. Mass accuracies and retention-time extraction windows were automatically determined by DIA-NN during the calibration step.

MaxQuant (version 1.6.3.4; Max-Planck-Institute of Biochemistry, Planegg, Germany) was used for protein identification and quantification for data acquired in DDA mode. The precursor mass tolerances were set to 20 ppm in the first search and 4.5 ppm in the main search, and the fragment mass tolerance was set to 20 ppm.

For all data searches, the proteome database from *Homo sapiens* (downloaded from Uniprot on 8^th^ of December 2020) was used for peptide and protein identification. Cysteine carbamidomethylation was set as a fixed modification and methionine oxidation was set as a variable modification. The precursor FDR was set as 1 %.

Proteins were filtered to include only those quantified in at least three of the six replicates (hexaplicates) in at least one condition. Statistical comparison of protein abundance between conditions was performed using a Student’s t-test and fold-change analysis. P-values from two-sample t-tests were adjusted using a permutation-based false discovery rate approach (FDR < 0.01 for targeted and untargeted biotinylation or FDR <0.05 for GCPII pulldown).

Complete lists of identified proteins and their corresponding fold-change are available online as Supplementary Data S1–S3. The overlap analysis of resulting proteins datasets was performed using BioVenn.^19^

**SDS-PAGE and Western blotting**

Protein elutions were analyzed by SDS-PAGE, employing Protein Dual Color Standards for molecular weight determination. The stacking gel was removed, and the separating gel was transferred onto a nitrocellulose membrane of the same size (wet electroblotting: 100 V/1 h). Membranes were blocked with casein blocker in TBS (Thermo Scientific) at room temperature for 1 h and incubated in primary antibodies overnight at 4 °C. Then, membranes were washed three times with PBS containing 0.05% Tween 20 (PBST) and incubated with secondary antibodies for 1.5 h at room temperature. Finally, the blots were washed three times with PBST and once with PBS to remove free antibodies. Images were acquired using near-infrared laser scanning on Odyssey CLx® Imaging System (LI-COR®) with two-color detection at 700 nm and 800 nm, following the manufacturer’s instructions**.** Finally, the images were converted to grayscale using the GIMP software.

The following antibodies diluted in casein blocker in TBS were used for immunodetection: the primary mouse antibody GCP-04 (anti-GCPII)^20^ (200 ng/ml), IRDye 800CW Streptavidin (LI-COR^®^ 926-32230; 1:15,000), rabbit antibody CD97/ADGRE5 (Sino Biological, 11280-T54, 1:500), rabbit antibody BCAM (Sino Biological, 10238 -RP02, 1:500), rabbit antibody CD151 (Sino Biological, 200150-T32, 1:500), rabbit antibody CD9 (Abcam, ab92726, 1:500), mouse antibody Dystroglycan/DAG1 (ProteinTech, 66735-1-Ig, 1:5000), rabbit antibody NCAM1 (Sino Biological, 10673-T54, 1:500), rabbit antibody PTK7 (Sino Biological, 204330-T08, 1:500), rabbit antibody Glast/SLC1A3 (Invitrogen, PA5-72895, 1:500), rabbit antibody CD98/SLC3A2 (Sino Biological, 12206-T62, 1:500), rabbit antibody Glut1/ SLC2A1 (Cell Signaling Technology, 12939, 1:1,000), IRDye 800CW Goat anti-Mouse Secondary Antibody (LI-COR^®^, 926-32210; 1:15,000), IRDye 800CW Goat anti-Rabbit Secondary Antibody (LI-COR^®^, 926-32211; 1:15,000).

**Pull-down/immunoprecipitation of GCPII and adjacent proteins from U-251 MG–GCPII lysate**

Each sample contained 8 million U251 MG–GCPII. Cells were lysed by sonication 2x 15 s on ice using an Elmasonic S 30 in 50 mM Tris-HCl, pH 7.4, 100 mM NaCl, 1.5% (w/w) *N*-Dodecyl b-D-maltoside, and 1 tablet of Complete protease inhibitor per 10 mL. The samples were left 1 h on ice and then centrifuged at 10,000 rcf for 10 min at 4 °C. The resulting cell lysate was measured by Bradford protein assay and diluted in TBS to a final protein concentration of 0.73 mg/mL.

First, 200 μL of 500 nM **iBody** **5** (conjugated with **x**GCPII and biotin) or **iBody 6** (conjugated with biotin) in 20 mM Tris-HCl, pH 7.4, 150 mM NaCl, 0.1% Tween 20 (TBST) was bound onto 40 μL of Streptavidin Agarose Ultra Performance (VectorLabs, N-1000) for 1 h at 4 °C. The beads were pelleted at 1,000 rcf for 1 min at 4 °C and washed 3x with 1 mL TBST, mixed with 750 μL of the U-251 MG–GCPII cell lysate, and incubated overnight at 4 °C on a rotisserie. The resin was washed 6x with 1 mL TBS. After the final wash, proteins were eluted with reducing SDS sample buffer and heated to 95 °C for 5 min. Protein elutions were analyzed using LC-MS/MS. The experiment was conducted in pentaplicates.

**References**

1 Beranová, J. *et al.* Tris-(Nitrilotriacetic Acid)-Decorated Polymer Conjugates as Tools for Immobilization and Visualization of His-Tagged Proteins. *Catalysts* **9**, 1011 (2019).

2 Metternich, J. B. *et al.* Covalent Immobilization of (-)-Riboflavin on Polymer Functionalized Silica Particles: Application in the Photocatalytic E→Z Isomerization of Polarized Alkenes. *Chemistry* **24**, 4228–4233 (2018). <https://doi.org/10.1002/chem.201800231>

3 Geri, J. B. *et al.* Microenvironment mapping via Dexter energy transfer on immune cells. *Science* **367**, 1091–1097 (2020). <https://doi.org/10.1126/science.aay4106>

4 Šubr, V., Kostka, L., Plicka, J., Sedláček, O. & Etrych, T. Highly Effective Synthetic Polymer-Based Blockers of Non-Specific Interactions in Immunochemical Analyses. *Polymers (Basel)* **16** (2024). <https://doi.org/10.3390/polym16060758>

5 Subr, V. & Ulbrich, K. Synthesis and properties of new N-(2-hydroxypropyl)-methacrylamide copolymers containing thiazolidine-2-thione reactive groups. *React Funct Polym* **66**, 1525–1538 (2006). <https://doi.org/10.1016/j.reactfunctpolym.2006.05.002>

6 Ishitake, K., Satoh, K., Kamigaito, M. & Okamoto, Y. Stereogradient polymers formed by controlled/living radical polymerization of bulky methacrylate monomers. *Angew Chem Int Ed Engl* **48**, 1991–1994 (2009). <https://doi.org/10.1002/anie.200805168>

7 Subr, V. *et al.* The role of the biotin linker in polymer antibody mimetics, iBodies, in biochemical assays. *Polym Chem-Uk* **12**, 6009–6021 (2021). <https://doi.org/10.1039/d1py00707f>

8 Gilmore, J. M., Scheck, R. A., Esser-Kahn, A. P., Joshi, N. S. & Francis, M. B. N-terminal protein modification through a biomimetic transamination reaction. *Angew Chem Int Ed Engl* **45**, 5307–5311 (2006). <https://doi.org/10.1002/anie.200600368>

9 Crisalli, P. & Kool, E. T. Water-soluble organocatalysts for hydrazone and oxime formation. *J Org Chem* **78**, 1184–1189 (2013). <https://doi.org/10.1021/jo302746p>

10 Perrier, S., Takolpuckdee, P. & Mars, C. A. Reversible addition-fragmentation chain transfer polymerization: End group modification for functionalized polymers and chain transfer agent recovery. *Macromolecules* **38**, 2033–2036 (2005). <https://doi.org/10.1021/ma047611m>

11 Šácha, P. *et al.* iBodies: Modular Synthetic Antibody Mimetics Based on Hydrophilic Polymers Decorated with Functional Moieties. *Angew Chem Int Ed Engl* **55**, 2356–2360 (2016). <https://doi.org/10.1002/anie.201508642>

12 Neburkova, J. *et al.* Inhibitor-GCPII Interaction: Selective and Robust System for Targeting Cancer Cells with Structurally Diverse Nanoparticles. *Mol Pharm* **15**, 2932–2945 (2018). <https://doi.org/10.1021/acs.molpharmaceut.7b00889>

13 Rees, J. S., Li, X. W., Perrett, S., Lilley, K. S. & Jackson, A. P. Selective Proteomic Proximity Labeling Assay Using Tyramide (SPPLAT): A Quantitative Method for the Proteomic Analysis of Localized Membrane-Bound Protein Clusters. *Curr Protoc Protein Sci* **80**, 19.27.11–19.27.18 (2015). <https://doi.org/10.1002/0471140864.ps1927s80>

14 Oslund, R. *et al.* in *VIII EFMC International Symposium on Advances in Synthetic and Medicinal Chemistry* (Athens, Greece, 2019).

15 Erde, J., Loo, R. R. & Loo, J. A. Enhanced FASP (eFASP) to increase proteome coverage and sample recovery for quantitative proteomic experiments. *J Proteome Res* **13**, 1885–1895 (2014). <https://doi.org/10.1021/pr4010019>

16 Deutsch, E. *et al.* The ProteomeXchange consortium in 2020: enabling 'big data' approaches in proteomics. *Nucleic Acids Research* **48**, D1145–D1152 (2020). <https://doi.org/10.1093/nar/gkz984>

17 Perez-Riverol, Y. *et al.* The PRIDE database resources in 2022: a hub for mass spectrometry-based proteomics evidences. *Nucleic Acids Research* **50**, D543–D552 (2022). <https://doi.org/10.1093/nar/gkab1038>

18 Demichev, V., Messner, C. B., Vernardis, S. I., Lilley, K. S. & Ralser, M. DIA-NN: neural networks and interference correction enable deep proteome coverage in high throughput. *Nat Methods* **17**, 41–44 (2020). <https://doi.org/10.1038/s41592-019-0638-x>

19 Hulsen, T., de Vlieg, J. & Alkema, W. BioVenn - a web application for the comparison and visualization of biological lists using area-proportional Venn diagrams. *BMC Genomics* **9**, 488 (2008). <https://doi.org/10.1186/1471-2164-9-488>

20 Sácha, P. *et al.* Expression of glutamate carboxypeptidase II in human brain. *Neuroscience* **144**, 1361–1372 (2007). <https://doi.org/10.1016/j.neuroscience.2006.10.022>
